# Target Tissue Identification Based on Image Processing for Regulating Automatic Robotic Lung Biopsy Sampler: Onsite Phantom Validation

**DOI:** 10.3390/s26051723

**Published:** 2026-03-09

**Authors:** Maria Monserrat Diaz-Hernandez, Gerardo Ramirez-Nava, Isaac Chairez

**Affiliations:** 1 Tecnologico de Monterrey, School of Engineering and Sciences, Guadalajara 45210, Mexico; mariad@tec.mx; 2 Tecnologico de Monterrey, Institute of Advanced Materials for Sustainable Manufacturing, Guadalajara 45210, Mexico; gerardo.j.ramirez@tec.mx

**Keywords:** image processing, medical robots, multiple robot configurations, automatic biopsy sampling, radio-pharmaceutical

## Abstract

Cancer is one of the global health problems that affects millions of people every year. Biopsies are among the standard methods for detecting and confirming a cancer diagnosis. Performing this study manually poses several challenges due to tissue movement and the difficulty of precisely locating the target, as is often the case in lung biopsies. This study presents the design and implementation of an autonomous image processing algorithm included in a closed-loop controller that drives the activity of a multi-degree-of-freedom (six) robotic manipulator that performs emulated tissue biopsies. A realistic lung motion emulator, based on a two-degree-of-freedom robotic device with a photon emitter (to simulate radiopharmaceutical identification of cancerous tissue), was used to test the proposed automatic biopsy collector. Applying image processing to detect cancer tissue enables the identification of the centroid and tumor boundaries. Using the detected centroid coordinates, the reference trajectory of the end effector (biopsy needle) was automatically determined. A finite-time convergent controller was implemented to guide the robotic manipulator’s motion towards the tumor position within a specified time window. The controller was evaluated using a digital twin representation of the entire robotic system and using an experimental device working on the simulated mobile tumor emulator. Evaluation of simulated tumor detection and reference trajectory tracking effectiveness was used to validate the operation of the proposed automatic robotic lung biopsy sampler. The application of the controller allows one to track the position of the emulated tumor with a deviation of 0.52 mm and a settling time of less than 1 s.

## 1. Introduction

Cancer is a disease characterized by uncontrolled cellular proliferation and dissemination throughout the body [1], making it one of the most significant global health challenges. The importance of addressing cancer lies in its prevalence and in the potential to improve early detection, treatment, and recovery, thereby enhancing overall quality of life [2]. According to the World Health Organization, lung cancer accounted for approximately 1.80 million of the 10 million cancer-related deaths reported in 2020 [1]. One of the primary reasons for mortality in lung or breast cancer is late-stage diagnosis [3]. The diagnosis is primarily established through a sequence of stages that include biochemical and/or imaging techniques. These initial analyzes are complemented by the collection of tissue from the neoplasm (tumor), which is usually performed manually by an expert. Note that collecting tissue from a tumor is crucial for effective cancer diagnosis, as other methods are not definitive until the pathologist performs a tissue study.

One of the crucial challenges in several cancer diagnoses is that the tumor may change its position depending on tissue or organ motion. The time associated with the movement is technically known as intrafractional tumor motion [4]. The most well-known type of tumor that can change its position within the body is a lung tumor. This motion depends on the patient’s respiratory rate and anatomy [5]. Tumor movement presents one of the most challenging conditions for performing a precise diagnosis. This is particularly remarkable, as early detection and understanding of tumor pathogenesis allow for the selection of an effective therapy for the patient [3].

Imaging research, laboratory tumor markers, and cytopathological examination are among the tools used to accurately diagnose different types of cancer. Today, medical imaging is becoming the first approach to cancer diagnosis [6]. However, when the tumor moves during the study, it can hinder the true nature and location of the tumor tissue. Indeed, the lack of precise tumor tissue location must be compensated for by a radiotherapist or medical doctor who spends a lot of time correcting the acquired images [7].

In addition, when tumor characteristics indicate a malignant neoplasm, it is necessary to confirm the diagnosis by collecting a sample of the target tissue. This procedure is called biopsy sampling. Biopsy is the most common procedure for detecting cancer in tissue samples collected from the human body. The collected tissue is analyzed by a pathologist using microscopic methods, who determines the tumor’s characteristics and establishes the basis for treatment [8,9,10]. The most prevalent biopsy methods are incisional, excisional, and needle biopsies [11]. In clinical practice, such image-guided diagnostic and interventional procedures can be supported by a range of medical imaging modalities, including ultrasound (US), conventional radiography (X-ray), computed tomography (CT), and nuclear medicine techniques such as positron emission tomography (PET) and single-photon emission computed tomography (SPECT) [12].

In nuclear imaging modalities, the emission of ionizing radiation from radiotracers within the patient is not directly observed; instead, high-energy photons are detected by dedicated sensor systems and converted into electrical signals, which are subsequently processed through reconstruction algorithms to generate spatially resolved images representing the radiotracer distribution [13,14]. Hybrid imaging systems, such as PET/CT and SPECT/CT, integrate this functional information with anatomical data, enabling accurate spatial localization of regions of interest within complex anatomical environments [15,16]. This fusion of functional and structural imaging enhances target delineation and spatial contextualization, which are critical for diagnostic assessment, treatment planning, and image-guided biopsy procedures.

Despite their numerous benefits, manual biopsy procedures have several shortcomings, including limited stability and accuracy, and a high degree of dependence on the operator’s expertise [17]. To address these issues, various technologies (including robotic systems) have been introduced. These systems, through their precise mechanisms and tumor location capabilities, improve precision and provide greater stability during biopsy procedures [18,19].

Robotic technologies are becoming increasingly important in lung biopsy because they aim to improve targeting precision, procedural reproducibility, and patient safety, particularly in small, deep, or difficult-to-reach pulmonary lesions. From a clinical perspective, robotic systems can be grouped into two complementary paradigms with distinct technical and clinical advantages: (i) robotic-assisted bronchoscopy, which enables controlled endoluminal navigation toward peripheral targets and has been shown to provide tissue suitable for downstream molecular marker analysis in primary lung cancer [20], while also enabling workflow optimization, such as multi-site biopsy within a single procedure using shape-sensing robotic bronchoscopy platforms [21]; and (ii) image-guided percutaneous robotic biopsy (CT or PET/CT-guided), where robotic needle positioning can improve trajectory stability and targeting accuracy and has been systematically reviewed across available clinical solutions for percutaneous lung biopsies [22], including PET/CT-guided robotic assistance reports describing enhanced accuracy and clinical outcomes [23]. In parallel, emerging research prototypes such as flexible biopsy robots integrating force sensing are being developed to increase controllability and safety during deep lung examination [24], consistent with recent interventional diagnostic reviews that identify robotic biopsy as a key direction within novel strategies for lung cancer diagnosis [25].

Various robotic technologies have been developed to perform biopsies in conjunction with medical imaging systems, aiming to improve early detection and timely diagnosis. Some notable examples include

- Biobot Surgical’s iSR’obot Mona Lisa, a robotic system specifically designed for prostate biopsy procedures [26];
- ROBIO, developed by XACT Robotics, enables needle-based percutaneous procedures, including biopsies and ablations—in multiple anatomical regions, integrating real-time imaging and robotic guidance to achieve accurate targeting of the lesion and tissue sampling [27];
- The ANT-C robot by NDR Medical Technologies is an advanced robotic assistant for minimally invasive procedures in interventional radiology, supporting precise needle placement during biopsies and ablations guided by imaging modalities such as CT or ultrasound [28].

One of the main limitations of the previous automatic biopsy equipment is the lack of automation in tissue collection. Effective biopsy procedures require a feedback system that integrates insertion and extraction forces with needle position to prevent tissue tearing, minimize the risk of spreading potentially cancerous cells, and reduce patient harm. Therefore, this study proposes the development of a system for automated tissue collection during lung biopsy, incorporating the simulation of the procedure using medical nuclear imaging.

The primary contribution of this study is a robotic-arm system designed to improve precision and efficiency in tissue collection during lung biopsy procedures. This system integrates advanced imaging techniques that combine nuclear medicine simulations, enabling real-time lesion localization and accurate navigation within complex lung structures. Using robotic assistance, the system aims to reduce human error, shorten procedure time, and ensure effective sample acquisition, ultimately improving diagnostic accuracy and patient outcomes. In addition, incorporating artificial intelligence for image analysis and trajectory planning further enhances the system’s capabilities, significantly advancing minimally invasive pulmonary interventions.

This paper is organized into four sections to present the development, validation, and potential impact of the proposed robotic-assisted system for lung biopsy procedures. Section 2 describes the system’s design, including the robotic arm, XY positioning mechanism, integration with imaging modalities such as nuclear medicine and computed tomography, and the control algorithms used for trajectory tracking and operational workflow. Section 3 presents the experimental and simulation results, evaluating the accuracy, stability, and performance of the tissue collection system, together with a comparative analysis with existing techniques. Finally, Section 4 examines the implications of the results, discussing the system’s advantages, limitations, and potential areas for improvement, while providing guidance for future research and development.

## 2. Methodology

**Figure 1:**
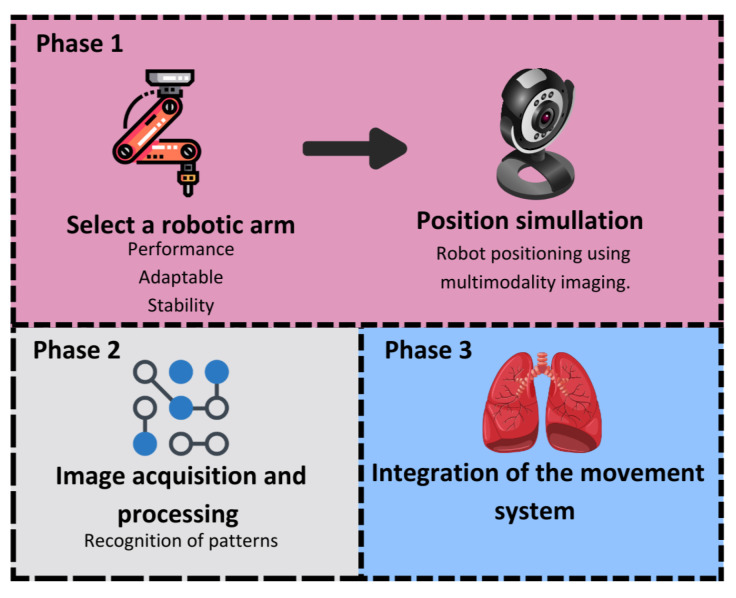
Methodology for implementing a robotic image tracking and processing system.

The methodology followed in this study was implemented in three key phases, as shown in Figure 1, progressing from validated mechanical design to the operation of an integrated robotic system comprising a robotic arm and a Cartesian device.

- Phase 1; Integration and simulation: The process begins with the selection of a robotic arm, such as the MyCobot 280 M5 (Elephant robotics, Shenzhen, China) chosen for its six degrees of freedom (DOF). This arm, combined with a 2D Cartesian robot, creates a flexible, high-precision system. A virtual twin of the robotic system was then developed, enabling simulation and visualization of the arm’s movements in a virtual environment.
- Phase 2; Image acquisition and processing: A spherical mechanism that generates oscillatory movements was designed to allow motion in two complementary planes, strategically positioning a matrix LED that creates detectable patterns for the camera system. This setup mimics various industrial and medical scenarios. Then, image-processing techniques were employed, involving a thresholding algorithm to isolate high-contrast areas and generate a distinct mask. The centroid of this mask, representing the target location, was identified as the critical point where the robotic arm’s end-effector must be positioned.
- Phase 3; In the third phase, both the simulation and the physical assembly were performed to test the functionality and performance of the combined system. Finite-time-convergent automatic control actions were implemented, enabling the robotic arm to effectively track and position itself relative to the targets, confirming the successful integration.

The automatic controller implementation involves planning the sequence of the sampler actions, including the design of a reference trajectory for the end-effector, and executing the biopsy procedure through four defined stages:

1. Positioning: The robot is positioned to allow access to the target tissue for sample collection.
2. Needle Insertion: The robot inserts the needle into the tumor to reach the target area. A force sensor is used to monitor the force applied during insertion to avoid damage to surrounding tissues.
3. Needle Retraction: Once the sample is collected, the robot retracts the needle and returns to the initial positioning stage.
4. Sample Retrieval and System Reset: The specialist retrieves the collected sample and moves the robot to its home (zero) position.

### 2.1. Integration of the Automatic Biopsy Sampler

**Figure 2:**
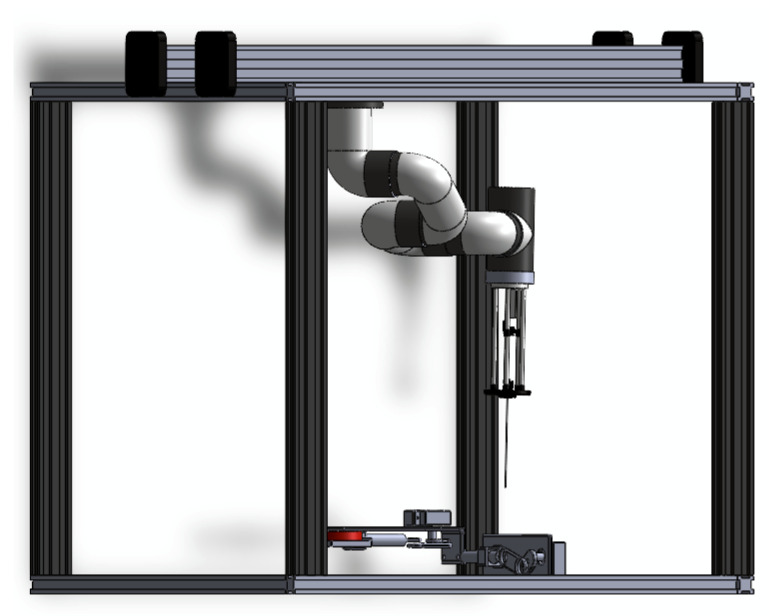
CAD model for developing the digital twin of the robotic system.

The most common complication of a biopsy, whether image-guided or manual, is a pneumothorax. The incidence of this complication ranges from 20% to 40%. This incidence increases with a decrease in nodule size, an increase in emphysema, and a reduction in the needle-to-skin angle [29]. In this context, robotic systems for needle positioning offer an ideal solution, ensuring superior precision, minimizing human error, and improving procedural outcomes. This section presents the design and implementation of the automated robotic biopsy sampler (Figure 2).

#### 2.1.1. Mechanical Design

A composite robotic system is proposed to perform the automatic biopsy sampling. The device integrates a six-degree-of-freedom robotic arm with a Cartesian XY carrier positioning mechanism. This configuration enables precise placement of the robotic arm near the tumor-like target identified by simulated emission imaging framework, ensuring that the needle is positioned perpendicular to the skin and oriented along the planned trajectory. The proposed system is illustrated in Figure 2.

For the XY positioning system, an Ender5 Plus printer (Creality, Shenzhen, China) was mobilized to construct the XY structure due to its ease of programming and compatibility with the overall composite system. In addition, the MyCobot 280 M5 (a six-degree-of-freedom robotic manipulator) with a working radius of 280 mm and joint movement ranging from +165 to −165 degrees [30] allows the needle to be placed with the necessary constraint that it be perpendicular to the patient’s skin. The biopsy sampling needle was attached to a self-designed basis that can carry a force sensor to estimate the needle contact with patient tissue.

**Figure 3:**
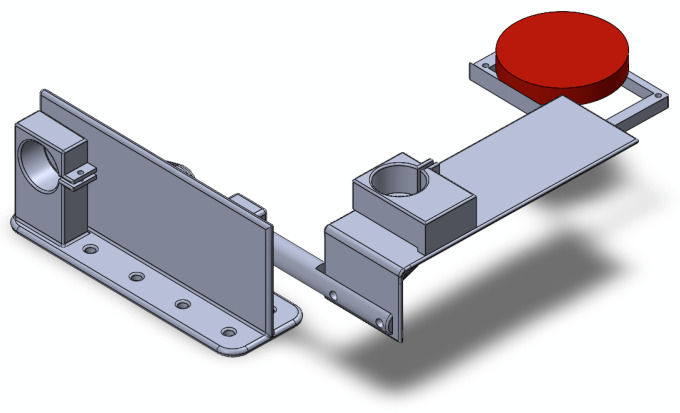
Mechanical configuration representation of the two-crank slider system generating tumor trajectories corresponding to the motion of a lung during a respiratory cycle. The red piece in the design represents the system that emulates photon emission from a target tumor using a radiopharmaceutical. This section of the device is controlled using a closed-loop control system based on a trajectory generator that corresponds to previously studied lung motions with tumoral tissue.

In addition, at the bottom of the robotic system, a two-degrees-of-freedom robotic system is configured to mobilize a lung phantom to test the proposed composite robotic device (Figure 3). The system is based on a couple of interconnected rod-crank devices, with an end effector that emits photons detectable by a photoreceptor in the visible spectrum. These devices were placed perpendicular to each other to allow for a spherical motion, similar to that observed in the lung during the respiratory cycle. This instrumented system is designed to emulate, at a conceptual level, the process of target localization based on radiation emission and detection. While the physical interactions governing the proposed system differ fundamentally from those involved in nuclear imaging, the underlying principle of detecting emitted radiation to generate a spatially resolved representation of a target is preserved. Consequently, the system does not replicate the physics or detection mechanisms of PET or SPECT instruments but rather abstracts the image formation process to study radiation-driven target identification and imaging within a controlled experimental framework.

#### 2.1.2. Electrical Instrumentation of the Composite Biopsy Automatic Sampler

Each section of the composite robotic biopsy sampler and the tumor emulator was instrumented to perform controlled movements according to the following strategy.

The XY stage at the top of the composite device was instrumented with linear actuators based on a stepper motor, featuring a front mounting flange and an attached screw/nut system. Each motor (NEMA 42, JSS motors, Jiangsu, China) was connected to a bidirectional power converter (DC-DC driver/chopper) with a 12-volt power source. An infrared linear distance sensor was instrumented to detect the base’s online position, where the MyCobot 280 M5 (Elephant robotics, Shenzhen, China) was attached. A digital processing board was used to control the motion of both actuators, implementing the developed controllers, and capturing data from the positioning sensors.

The robotic arm was not instrumented, except for its own integrated electronic system that drives each actuator per joint and its corresponding angular displacement sensor.

The dynamic tumor positioning emulator was equipped with two DC motors (RK-370SD-2470, Derry motors, Ningbo, China) and a bidirectional power converter (L298N, Derry motors, Ningbo, China), powered by a 6-volt voltage source. Once again, an independent digital processing board was used to regulate the motion of both actuators, implementing a closed-loop control strategy to modulate the spatial position of the emulated tumor tissue. All components of the robotic tumor tissue sampler were attached to a single computer (ATMega 2560 processing chip), which sends all commands to regulate the emulator, control the XY manipulator robots, and capture images from a webcam representing the acquisition process.

### 2.2. Control Design and Implementation

This section presents the control design details, its validation in a digital twin of the composite robotic device (including the emulated tumor tissue), and its implementation on the actual experimental robotic device.

The first stage of this process included obtaining the digital twin simulated in Matlab-Simulink^®^ 2024a and the actual device using the same original design in SolidWorks^®^ 2024. In addition, all mechanical elements in SolidWorks^®^ were adjusted to account for material yield to obtain similar mass and mechanical responses to shear and tangential stresses. In addition, most of the elements in the actual device were produced using additive manufacturing processes, which yield a device that corresponds to the original design. Furthermore, previous studies using this strategy confirmed that the dimensions of manufactured pieces did not deviate by more than 5% from those generated in the mechanical design. Moreover, when the manufactured material was originally fixed in the Solidwork’s^®^ design, the mass obtained from the pieces included in the final robotic device deviated no more than 5% from that obtained during the assembly. manufacturing procedure.

#### 2.2.1. Non-Singular Terminal Sliding Mode

The control of mobile robot manipulators, such as the one considered in this study, presents significant challenges due to uncertainties and disturbances, including payload variations and friction. In this context, sliding mode controllers (SMCs) have gained prominence in robotic control applications. When the goal is to reduce control gains and ensure the convergence of tracking errors in a finite time, a terminal sliding mode controller (TSM) is an effective choice [31]. However, this controller is known for its singularity-free performance at zero motion velocity. Non-singular terminal sliding mode control (NTSM) can be implemented to address singularity issues, offering robust performance while mitigating the mentioned limitations [31,32]. This study considers that NTSM is applied in a distributed strategy on the composite biopsy robotic sampler (CBRS) according to the following methodology: The dynamics of each joint of the eight joints (2 for the XY and 6 for the robotic arm) in the CBRS can be represented by (using the Euler-Lagrange equations):

(1) $$\begin{array}{cc} \frac{d}{dt}\xi_{1}^{<i>} & =\xi_{2}^{<i>}\\ \frac{d}{dt}\xi_{2}^{<2>} & =f^{<i>}\left(\xi_{1}^{<i>},\xi_{2}^{<i>}\right)+g^{<i>}\left(\xi_{1}^{<i>}\right)\tau_{i}+\sum_{j=1,j\neq i}^{8}g^{<i,j>}\left(\xi_{1}^{<j>}\right)\tau_{j}+\eta^{<i>}\left(\xi_{1},\xi_{2}\right) \end{array}$$

In this expression, $\xi_{1}^{<i>}$ is the position coordinate (linear for the XY and angular for the arm) of the i-th joint and $\xi_{2}^{<i>}$ is the corresponding velocity coordinate. The functions $f^{<i>}$, $g^{<i>}$, and $g^{<i,j>}$ represent drift, the effect of the own control $\tau^{<i>}$ on the i-th joint, and the effect of other controllers $\tau^{<j>}$ on the joint *j* on the dynamics of the i-th joint coordinate, respectively. The term $\eta^{<i>}$ characterizes the effect of the dynamics of all other joints (represented by the extended vectors $\xi_{1}={\left[\xi_{1}^{<1>},\ldots ,\xi_{1}^{<8>}\right]}^{⊤}$ and $\xi_{2}={\left[\xi_{2}^{<1>},\ldots ,\xi_{2}^{<8>}\right]}^{⊤}$) on the dynamics of the *i*-th joint.

The control design considers that $f^{<i>}$, $g^{<i>}$, and $g^{<i,j>}$ are known using the modeling strategies mentioned above, while $\eta^{<i>}$ is uncertain but satisfies the following assumption:

**Assumption** **1.** *The admissible class of uncertainties $\eta^{<i>}$ is characterized by the following set:*

(2) $$\Psi =\left\{\eta^{<i>}|\left|\eta^{<i>}\right|\leq \eta_{0}^{<i>}+\eta_{1}^{<i>}∥\xi ∥\right\}$$

*where $\xi ={\left[\xi_{1}^{⊤},\xi_{2}^{⊤}\right]}^{⊤}$ and $\eta_{0}^{<i>}$, $\eta_{1}^{<i>}$ two positive scalars.*

The control design problem addressed in this study involves mobilizing the CBRS to achieve a configuration within a finite time, enabling the robotic device to perform biopsy sampling while accounting for the identification of the target position via an automatic video processing strategy.

Assume that the target pose in the task space to take a biopsy is defined by $\rho^{*}$, a six-dimensional vector that includes position $\rho_{p}^{*}$ and orientation $\rho_{o}^{*}$, both three-dimensional vectors. Following a multidimensional interpolation method [33], the time-dependent, twice-differentiable function $\rho^{d}\left(t\right)$ (with bounded second derivative with respect to time) satisfies $∥\rho^{d}\left(T\right)-\rho^{*}∥\leq \varepsilon$ with ε an admissible distance between the desired position and the one obtained with the interpolation results.

Defining the position tracking error for the i-th joint as $\Delta_{a}^{<i>}=\rho^{*,<i>}-\xi_{1}^{<i>}$, its dynamics satisfies

(3) $$\begin{array}{cc} \frac{d}{dt}\Delta_{a}^{<i>} & =\Delta_{b}^{<i>}\\ \frac{d}{dt}\Delta_{b}^{<i>} & =\frac{d^{2}}{dt^{2}}\rho^{*,<i>}-f^{<i>}\left(\xi_{1}^{<i>},\xi_{2}^{<i>}\right)-\\ & g^{<i>}\left(\xi_{1}^{<i>}\right)\tau_{i}-\sum_{j=1,j\neq i}^{8}g^{<i,j>}\left(\xi_{1}^{<j>}\right)\tau_{j}-\eta^{<i>}\left(\xi_{1},\xi_{2}\right) \end{array}$$

The design of the NTSM control requires the introduction of an auxiliary variable *s* (usually called a sliding surface) characterized as follows [34].

(4) $$s^{<i>}=\Delta_{b}^{<i>}+\alpha^{<i>}{\left(\Delta_{a}^{<i>}\right)}^{\gamma^{<i>}}+\beta^{<i>}{\left(\Delta_{a}^{<i>}\right)}^{\vartheta^{<i>}}sgn\left(\Delta_{a}^{<i>}\right)$$

where $\alpha^{<i>}>0$, $\beta^{<i>}>0$, $0<\gamma^{<i>}<1$, $0<\vartheta^{<i>}$, $\gamma^{<i>}=\frac{q^{<i>}}{p^{<i>}}$ where $q^{<i>}$ and $p^{<i>}$ are positive odd integers.

The time derivative of the surface $s^{<i>}$ can be estimated (using the Dini formalism) as follows:

(5) $$\begin{array}{c} \frac{d}{dt}s^{<i>}=\frac{d}{dt}\Delta_{b}^{<i>}+\alpha^{<i>}\gamma^{<i>}{\left(\Delta_{a}^{<i>}\right)}^{\gamma^{<i>}-1}\Delta_{b}^{<i>}+\;\\ \beta^{<i>}\vartheta^{<i>}{\left(\Delta_{a}^{<i>}\right)}^{\vartheta^{<i>}-1}\Delta_{b}^{<i>}sgn\left(\Delta_{a}^{<i>}\right) \end{array}$$

Taking into account that the control action $\tau_{i}$ is proposed to satisfy

(6) $$\begin{array}{c} g^{<i>}\left(\xi_{1}^{<i>}\right)\tau_{i}=\frac{d^{2}}{dt^{2}}\rho^{*,<i>}-f^{<i>}\left(\xi_{1}^{<i>},\xi_{2}^{<i>}\right)-\sum_{j=1,j\neq i}^{8}g^{<i,j>}\left(\xi_{1}^{<j>}\right)\tau_{j}+\\ \alpha^{<i>}\gamma^{<i>}{\left(\Delta_{a}^{<i>}\right)}^{\gamma^{<i>}-1}\Delta_{b}^{<i>}+\beta^{<i>}\vartheta^{<i>}{\left(\Delta_{a}^{<i>}\right)}^{\vartheta^{<i>}-1}\Delta_{b}^{<i>}sgn\left(\Delta_{a}^{<i>}\right)+\;\\ K_{a}^{<i>}sgn\left(s^{<i>}\right)+K_{b}^{<i>}{\left(s^{<i>}\right)}^{\lambda^{<i>}} \end{array}$$

Then, the right-hand side of $\frac{d}{dt}s^{<i>}$ transforms to

(7) $$\begin{array}{c} \frac{d}{dt}s^{<i>}=-\eta^{<i>}\left(\xi_{1},\xi_{2}\right)-K_{a}^{<i>}sgn\left(s^{<i>}\right)-K_{b}^{<i>}{\left(s^{<i>}\right)}^{\lambda^{<i>}} \end{array}$$

Using the following energetic function for the entire robotic device is $V:=\sum_{i=1}^{8}V^{<i>}\left(s^{<i>}\right)$ with $V^{<i>}\left(s^{<i>}\right)=0.5{\left(s^{<i>}\right)}^{2}$, it is possible to prove that its derivative satisfies:

(8) $$\begin{array}{c} \frac{d}{dt}V^{<i>}\left(s^{<i>}\right)=s^{<i>}\frac{d}{dt}s^{<i>} \end{array}$$

Using the expression in (7) leads to

(9) $$\begin{array}{c} \frac{d}{dt}V^{<i>}\left(s^{<i>}\right)\leq -\left(K_{a}^{<i>}-\left|\eta^{<i>}\left(\xi_{1},\xi_{2}\right)\right|\right)\left|s^{<i>}\right|-K_{b}^{<i>}{\left(s^{<i>}\right)}^{\lambda^{<i>}+1} \end{array}$$

Assuming that vectors of position and velocity for the robotic device can be measured, select $K_{a}^{<i>}=\eta_{0}^{<i>}+\eta_{1}^{<i>}∥\xi ∥+K_{a,0}^{<i>}$. Then,

(10) $$\begin{array}{c} \frac{d}{dt}V^{<i>}\left(s^{<i>}\right)\leq -2^{1/2}K_{a,0}^{<i>}{\left(V^{<i>}\left(s^{<i>}\right)\right)}^{1/2}\\ -2^{(\lambda^{<i>}+1)/2}K_{b}^{<i>}{\left(V^{<i>}\left(s^{<i>}\right)\right)}^{(\lambda^{<i>}+1)/2} \end{array}$$

Using the inequality presented in [35,36], it is possible to confirm that the origin is a robust attractive equilibrium point of finite-time for the dynamics of tracking error with a settling function given by

(11) $$T=\sum_{i=1}^{8}\left(\frac{1}{0.5\left(2^{1/2}K_{a,0}^{<i>}\right)}+\frac{1}{\left(2^{(\lambda^{<i>}+1)/2}K_{b}^{<i>}\right)\left(\lambda^{<i>}-1)/2\right)}\right)$$

#### 2.2.2. Tests of the Controller on a Digital Twin of the CBRS

This study aimed to test the proposed NTSM controller on a digital twin of the CBRS system. The digital twin was used to provide online, efficient, and cost-effective assessment services in various technical fields, particularly for trajectory-tracking analysis [37]. The proposed digital twin was originally designed in SolidWorks^TM^ corresponding to the model observed in Figure 2. All elements of the digital twin were designed to match the physical parameters of the actual device, with the aim of achieving a realistic implementation of the controller and its corresponding gain adjustments.

The model obtained was transferred to the MATLAB/Simulink system^TM^ and evaluated in Simulink Toolbox^TM^. This model was used to validate the tracking and convergence properties of deviation errors $\Delta_{a}^{<i>}$. The integration of a digital twin and the dynamic simulation of control actions enabled the simulation of trajectories and the testing of different control strategies to evaluate and identify the most energy-efficient solution, while minimizing joint vibrations in the robot. This is crucial as even small vibrations can cause the needle to deviate from the target angle.

### 2.3. Automatic Identification of Target Tissue and Reference Trajectories Generation

The image acquisition strategy was proposed to track the desired position of the emulated tumor tissue.

A webcam sensitive to photons in the visible range was employed within a simulated emission imaging framework to enable real-time image acquisition, allowing online monitoring and accurate localization of tumor-like target motion within the CBRS workspace.

#### 2.3.1. Phantom of Tumor Tissue Movement in Human Lungs

The lung phantom was constructed in modular parts with a mechanical system for controlled motion; within the image-guided framework, the system can mimic an emission process that can be registered and recorded for target localization and tracking evaluation.

A model was designed to simulate the motion of the tumor, taking into account the physiological characteristics of the breathing cycle. The emulated tumor was proposed to perform a controlled motion following a regular breathing cycle, represented by three breathing movements: craniocaudal (up and down), which is the movement of the diaphragm during breathing [38], anteroposterior (forward and backward) and laterolateral (side to side), which is the expansion of the rib cage [39].

The tumor-like trajectory in the lung can be modeled as a hysteresis loop, whose shape depends on the patient’s respiratory mechanics. In the article “Open Source 3D Printed Lung Tumor Movement Simulation for Radiotherapy Quality Assurance” by [40], it is reported that the lungs are in constant motion and that tumor position changes continuously; therefore, respiratory-induced tumor motion must be considered in lung cancer research.

**Figure 4:**
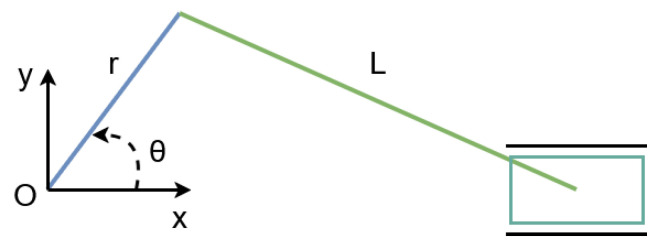
Piston–connecting rod–crank mechanism used to model tumor motion.

To replicate this hysteretic motion, the kinematic model of a piston–connecting rod–crank mechanism was adopted. The mechanism is shown in Figure 4, and the piston position with respect to the origin is described by:

(12) $$x\left(\theta \right)=r\cos\left(\theta \right)+\sqrt{\;l^{2}-r^{2}\sin^{2}\left(\theta \right)\;}.$$

To follow the prescribed hysteresis trajectory, it is necessary to compute the crank angle θ from the desired piston position. Let $x^{*}$ denote the desired (target) piston position. Then, the position tracking error can be defined as:

(13) $$\Delta x=x^{*}-\left(r\cos\left(\theta \right)+\sqrt{\;l^{2}-r^{2}\sin^{2}\left(\theta \right)\;}\right).$$

Solving Equation (12) for θ yields

(14) $$\theta =\arccos\;\left(\frac{x^{*2}-l^{2}+r^{2}}{2rx^{*}}\right).$$

**Figure 5:**
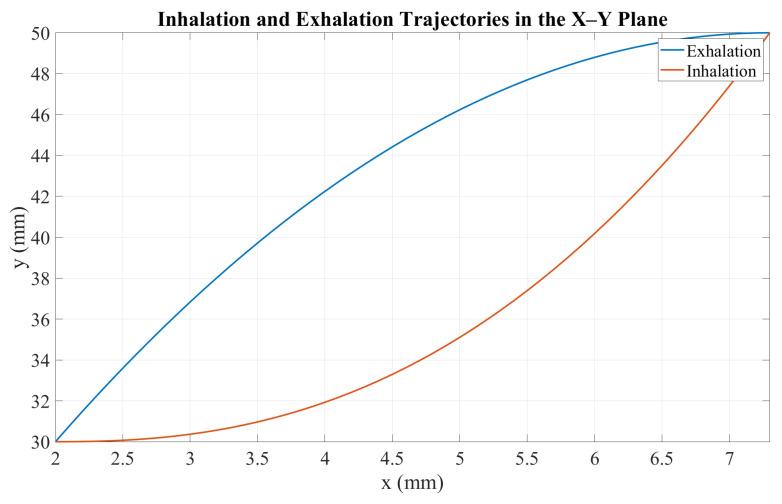
Inhalation and exhalation trajectories in the X-Y plane.

**Figure 6:**
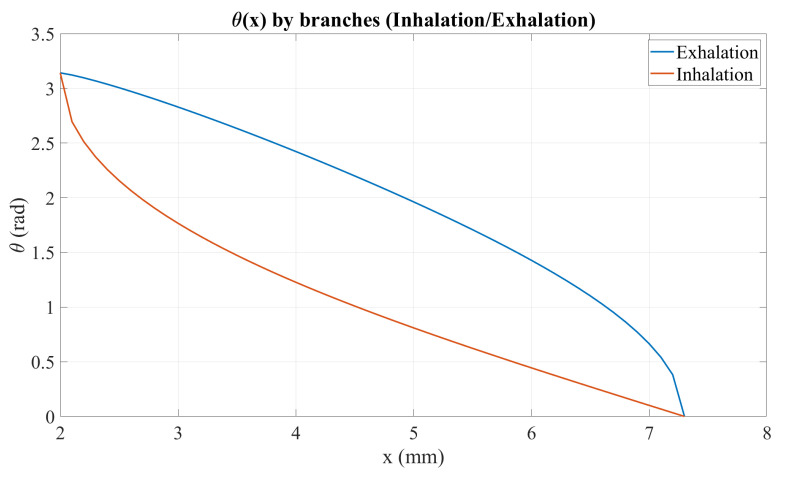
Angular profile θ(x) for inhalation and exhalation.

**Figure 7:**
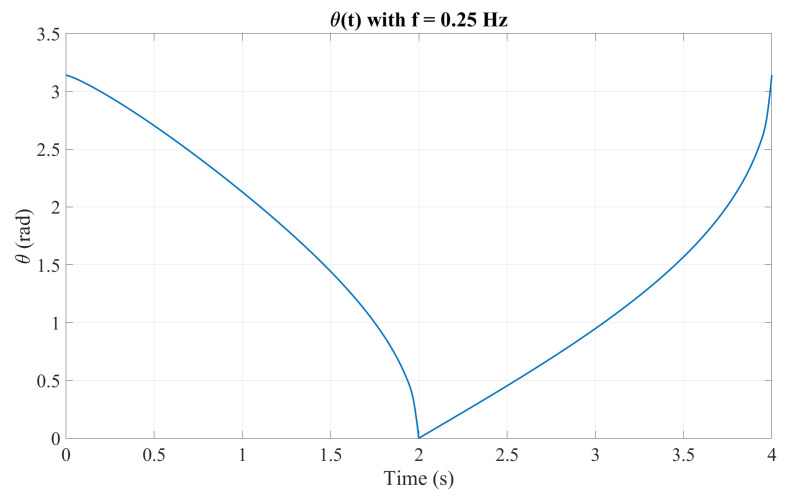
Angular displacement θ(t) at 0.25Hz.

To generate the inhalation and exhalation profiles, a Matlab 2024a script was developed to define the tumor-like motion within the ranges x∈[2,7]mm and y∈[30,50]mm. The mechanism parameters were set to r=10mm and l=40mm. Figure 5 shows the resulting X-Y trajectory over one breathing cycle, while Figure 6 presents the corresponding angular profile θ(x) derived from the hysteresis curve. Finally, Figure 7 shows the angular trajectory θ(t) required for the motor to reproduce the target motion at a breathing frequency of 0.25Hz.

The encoder reference signal was obtained by converting the motor shaft angle into encoder counts according to:

(15) $$counts=\left(\frac{\theta }{2\pi }\right)\;1632.67.$$

Each reference count value was sent to an Arduino Mega to drive the motors. A PD controller was implemented on the Arduino Mega to regulate the axis motion, where the control input was computed from the position tracking error $e\left(t\right)=counts_{\mathrm{ref}}\left(t\right)-counts\left(t\right)$ and its derivative $\dot{e}\left(t\right)$ as:

(16) $$u\left(t\right)=K_{p}e\left(t\right)+K_{d}\dot{e}\left(t\right).$$

#### 2.3.2. Simulated Emission Imaging Framework

**Figure 8:**
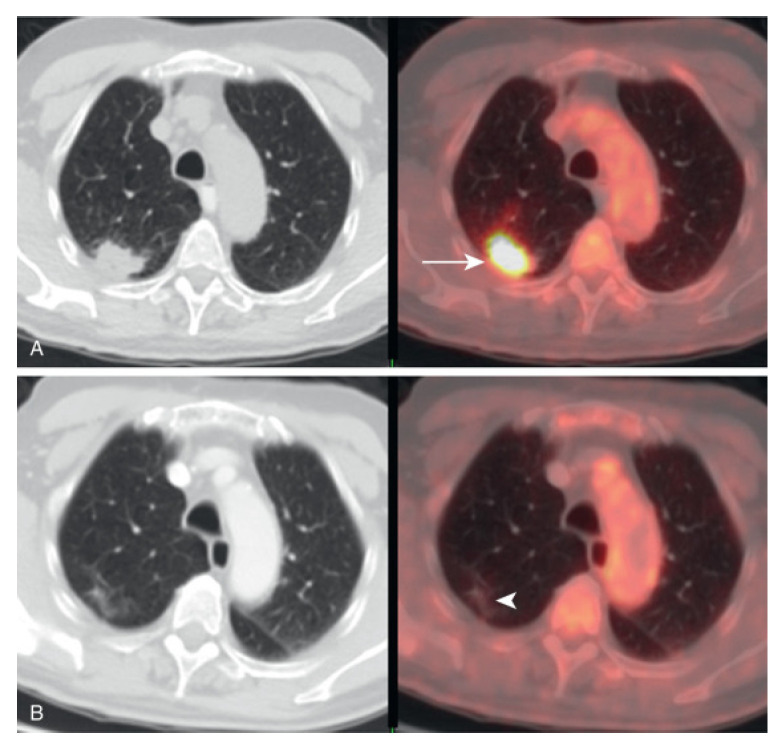
FDG PET/CT images of the lungs demonstrating areas of abnormal metabolic activity. (**A**) CT image (**left**) and PET/CT fusion image (**right**) highlight a hypermetabolic lesion (arrow) suggestive of malignancy. (**B**) Additional CT (**left**) and PET/CT fusion (**right**) show reduced metabolic activity in a different region (arrowhead). These images illustrate the combined anatomical and functional insights provided by PET/CT imaging in pulmonary evaluations. This image was obtained from [42].

To study image-guided target localization in a controlled environment, a tumor-like target was represented using an LED matrix, providing a reproducible emission source for image acquisition. Lung geometry was obtained from thoracic CT data using 3D Slicer, which enables segmentation of anatomical regions of interest, and was subsequently used to fabricate a lung phantom via additive manufacturing techniques. Different transparent materials, including clear polylactic acid (PLA), polyethylene terephthalate glycol-modified (PETG), clear resin, and thermoplastic polyurethane (TPU), were evaluated to determine their suitability for enabling the emitted signal to be registered by the imaging system. These materials were selected due to their low cost, widespread availability in manufacturing and prototyping laboratories, and compatibility with desktop-scale additive manufacturing systems, while providing varying degrees of optical transparency relevant to image formation (Figure 8). Mechanical properties, such as elasticity or tissue compliance, were not considered in the material selection, as they fall outside the scope of the present study (Table 1).

Image segmentation was then applied to partition the acquired images into pixel groups, allowing the extraction of features such as centroid localization, geometric characterization, and pattern recognition of the tumor-like target [41].

One method for global automatic thresholding is Otsu’s method. This algorithm is based on the principle that the gray level at which the variance between classes is maximized, or the variance within classes is minimized, is selected as the threshold [41,43]. The iterative approach provides an exact implementation of Otsu’s method, where the variance within each class is calculated as the weighted sum of the variances of the two classes, with their respective weights given by [43].

(17) $$\sigma_{within}^{2}\left(t\right)=\omega_{0}\left(t\right)\sigma_{0}^{2}+\omega_{1}\left(t\right)\sigma_{1}^{2}$$

where, $\omega_{0}\left(t\right)=\sum_{i=0}^{t}p_{i}$ and $\omega_{1}\left(t\right)=\sum_{i=t}^{L-1}p_{i}$  $\sigma_{0}^{2}$ is the variance of pixels in the background or below threshold, and $\sigma_{1}^{2}$ is the variance of pixels in the foreground or above threshold.

**Figure 9:**
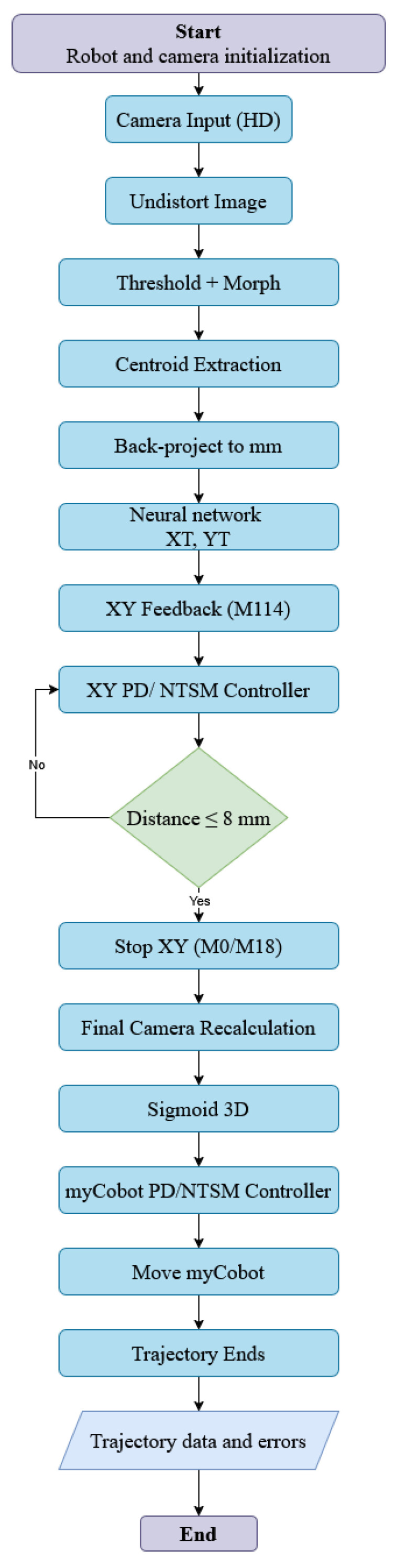
Flowchart describing the process of robot and camera initialization, image processing, and robotic movement.

Following the general sequence of steps outlined in Otsu’s method, the flow diagram in Figure 9 illustrates the process of segmenting and determining the centroid of images acquired from a lung emulator using a camera. Segmentation and image processing were implemented in MATLAB.

### 2.4. Integration of Mechanical and Image Acquisition System

The flow diagram in Figure 9 outlines the steps required to move the robot to the centroid of the tumor. This process integrates the XY coordinate system, the MyCobot robotic arm, and the Otsu method for image processing and centroid determination. The integrated system displays the relative position of the tumor tissue and the mobile robotic device in a working scenario for both the digital twin system and the experimental CBRS device.

The first stage consisted of defining the initial operating parameters for the camera, the XY mounting system, the myCobot manipulator, and the lung emulator. The camera parameters were obtained through a calibration procedure using the chest-table calibration pattern, and the resulting intrinsic values were used to configure the image acquisition model. The depth of the workspace was assumed to be constant at $Z_{c}=650\;\mathrm{mm}$, given that the lung emulator remains confined to a planar region relative to the optical axis of the camera. Subsequently, the XY mounting system (Ender 5 Plus) was calibrated according to its internal coordinate reference, assigning the origin at $X_{m}=180$ and $Y_{m}=180$, which served as the global coordinate frame for all subsequent transformations. Finally, myCobot was initialized to a predefined home position to ensure collision-free operation and prevent interference with the lung phantom.

The second stage involved acquiring the region of interest and extracting the centroid of the binary mask corresponding to the location of the target sample. Each captured frame was processed through an image-segmentation pipeline consisting of:

- RGB-to-grayscale conversion,
- contrast enhancement using dynamic range adjustment,
- Otsu thresholding,
- morphological opening and closing, and 
- hole filling.

From the resulting binary mask, the largest connected component was selected, and its centroid (u,v) in pixel coordinates was calculated. These coordinates of the image-plane were back-projected into metric coordinates using the pinhole camera model [44]. The normalized image coordinates were obtained as:

(18) $$x_{n}=\frac{u-c_{x}}{f_{x}},$$

(19) $$y_{n}=\frac{v-c_{y}}{f_{y}},$$

and the reconstructed metric coordinates on the workspace plane were computed as:

(20) $$X_{c}=x_{n}Z_{c},$$

(21) $$Y_{c}=y_{n}Z_{c}.$$

In the following stage, the metric coordinates $\left(X_{c},Y_{c}\right)$ were used as input to a neural network of forward regression [45]. This network was trained to learn the nonlinear mapping from camera-based coordinate estimates to real workspace coordinates $\left(X_{m},Y_{m}\right)$ of the XY mounting platform. The normalized camera-frame input was passed through the trained model, whose output corresponds to the corrected physical coordinates used for robotic positioning. The use of the neural network was motivated by the tilt of the platform, mounting offsets, and residual geometric distortions that cannot be accurately compensated for using analytical transformations alone. When the trained coordinates $X_{T},Y_{T}$ are calculated, they are rotated to compensate for the yaw mismatch:

(22) $$\Delta \psi =\psi_{\mathrm{real}}-\psi_{0}$$

(23) $$R_{z}\left(\Delta \psi \right)=\left[\begin{array}{cc} \cos(\Delta \psi ) & \sin(\Delta \psi )\\ -\sin(\Delta \psi ) & \cos(\Delta \psi ) \end{array}\right]$$

The compensated target is:

(24) $$\left[\begin{array}{c} \hat{X}\\ \hat{Y} \end{array}\right]=R_{z}\left(\Delta \psi \right)\left[\begin{array}{c} X_{T}\\ Y_{T} \end{array}\right]$$

The prediction is saturated in the robot’s admissible workspace.

The PD and NTSM controllers were implemented for motion in the XY plane, the workspace being constrained so that the position error of the final effector remained below 8 mm. The current position feedback was obtained using the M114 command, and the motion commands were executed in relative mode (G91) before returning to absolute mode (G90). When the target position was reached within a tolerance of 8 mm, the XY table was safely stopped using the commands M0 and M18, and control authority was transferred to the robotic arm.

After re-estimating the target position and computing the real yaw angle, a three-dimensional sigmoid trajectory was generated over a duration of T=8 s with a sampling period of dt=0.05 s:

(25) $$S\left(t\right)=X_{i}+\frac{1}{1+e^{\left[-c(t-T/2)\right]}}\left(X_{f}-X_{i}\right).$$

Independent sigmoid profiles were generated for X(t), Y(t), Z(t), and the angle of yaw. A minimum allowable height constraint of Z≥ 80 mm was enforced and a safety radius of 260 mm defined the admissible operational region. Subsequently, the Cartesian PD and NTSM controllers were applied to track the generated trajectory and to evaluate the robot’s inverse kinematics during the execution of the desired end-effector motion. Finally, all data are recorded such as reference trajectory (X,Y,Z), real trajectory (X,Y,Z), axis errors $\left(e_{x},e_{y},e_{z}\right)$ and 3D error norm that was estimated as:

(26) $$∥e∥=\sqrt{e_{x}^{2}+e_{y}^{2}+e_{z}^{2}}$$

## 3. Results

This section evaluates the performance of the proposed NTSM controller using a digital twin model and the experimental CBRS device. To assess improvements in tracking performance, an extended-state feedback controller was proposed for evaluation in both numerical and experimental studies. In addition, it presents the operation of the lung phantom, including the identification of its motion through image processing using the Otsu method for segmentation and centroid detection. The integration of both systems (robotic control and image processing) is demonstrated, showcasing their combined functionality for precise tumor localization and corresponding tracking.

### 3.1. Control Performance of Digital Twin

**Figure 10:**
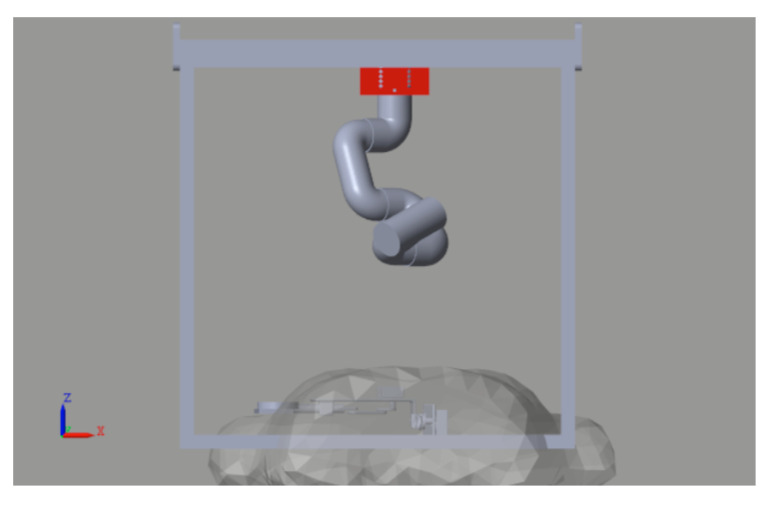
Digital twin of the CBRS in a realistic simulated workspace with the emulator of the tumoral tissue.

The digital twin model in Figure 10 presents the exported robotic manipulator operating within a defined workspace corresponding to the regular space in surgical rooms. The framework and surrounding environment are designed to facilitate online simulation interactions, enabling the validation of controller development on a realistic representation of the robotic system, which includes trajectory planning, CBRS kinematic analysis, and task execution (biopsy sampling).

To evaluate the proposed NTSM controller, we compared its performance with that of a distributed proportional-derivative (PD) controller. The PD controller combines feedforward and feedback control strategies, operating on the basis of current and predicted process conditions. Its output is a linear combination of the error signal and its derivative. By integrating the damping effect of proportional control with the predictive capabilities of derivative control, PD control effectively reduces fluctuations and anticipates process errors [46]. The mathematical representation of the PD control is given in Equation (27). Using a PD controller for robot manipulators enables consideration of joint flexibility, actuator dynamics, and friction, as proposed in [47].

(27) $$\tau_{i}=K_{p,i}\Delta_{a}^{<i>}+K_{d,i}\Delta_{b}^{<i>}$$

where $K_{p,i}$ is the proportional gain for the PD controller of the i-th joint, and $K_{d,i}$ is the derivative gain for the PD controller of the i-th joint. The selection of these gains was based on the method proposed in the study given by [48], and is presented in Table 2.

In addition, for comparison purposes, model predictive control (MPC) was considered in this study. This controller was defined using the Matlab Toolbox on Model Predictive Control using the following cost functional:

(28) $$J\left(\tau \left(\cdot \right)\right)=\int_{t=0}^{T}\left({∥\Delta_{a}∥}^{2}+{∥\Delta_{b}∥}^{2}+{∥\tau ∥}^{2}\right)dt$$

This specific cost function does not contain weighting matrices with the aim of performing a fair comparison with the suggested sliding mode base controller.

**Figure 11:**
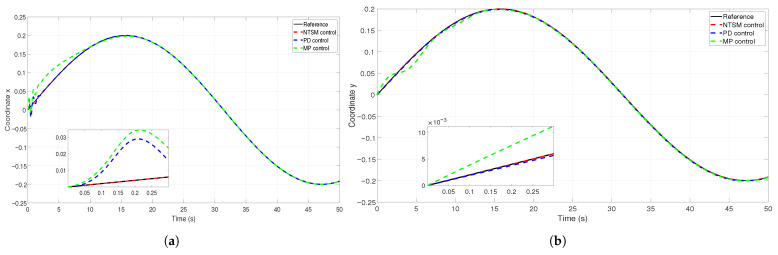
Temporal evolution of coordinates x and y. The reference signal (black solid line), the NTSM controller response (red dashed line), the PD controller response (blue dashed line), and the MPC controller outcome (green dashed line) are shown for each channel. The inset plots provide a zoomed view of the initial transient to highlight the differences in convergence and steady-state accuracy. (**a**) evolution of the coordinate x under the proposed collection of controllers. (**b**) evolution of the coordinate y under the proposed collection of controllers.

Figure 11 illustrates the tracking of reference trajectories for the XY system and for the set of multiple joints of a robotic manipulator. Each plot compares the reference trajectory (solid black line), the response of the NTSM controller (red dashed line), response of the PD controller (blue dashed line), and the results of a model predictive controller (green dashed line).

**Figure 12:**
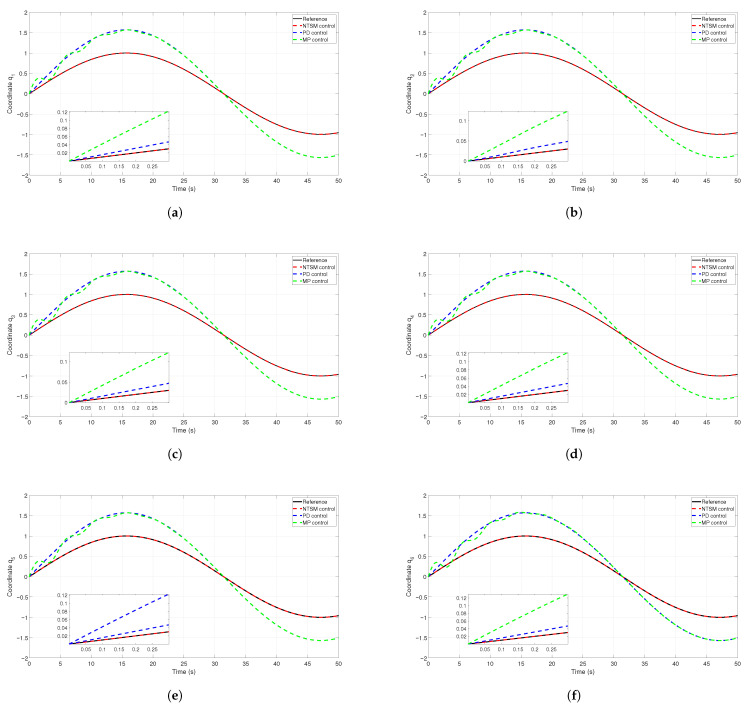
Temporal evolution of joint-space errors for joints 1–6. The reference signal (black solid line), the NTSM controller response (red dashed line), the PD controller response (blue dashed line), and the MPC controller outcome (green dashed line) are shown for each channel. The inset plots provide a zoomed view of the initial transient to highlight the differences in convergence and steady-state accuracy. (**a**) Coordinate $q_{1}$, (**b**) Coordinate $q_{2}$, (**c**) Coordinate $q_{3}$, (**d**) Coordinate $q_{4}$, (**e**) Coordinate $q_{5}$, and (**f**) Coordinate $q_{6}$.

The insets in each subplot show a zoomed view of the first 0.3 s to highlight the controllers’ behavior during the transient phase. The error (rad) is plotted over time (s) to better visualize the tracking performance and control precision in each scenario (Figure 12).

**Figure 13:**
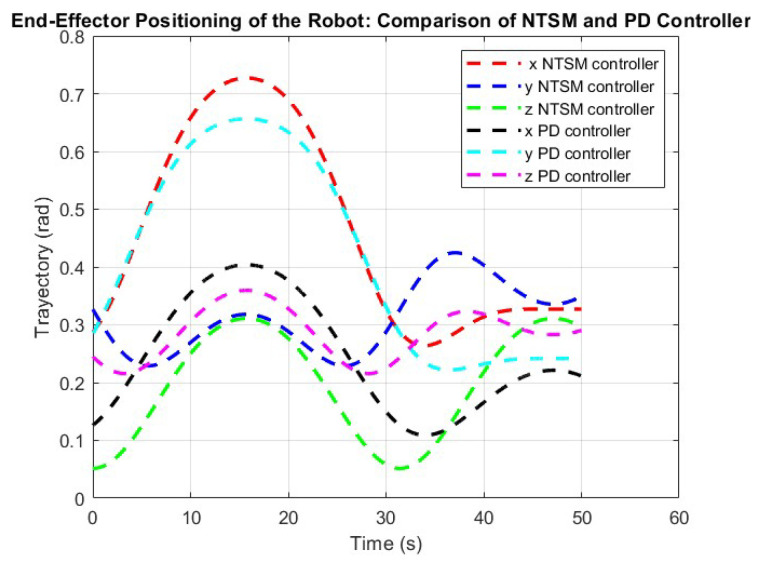
Trajectory tracking performance of the robot’s end-effector using NTSM and PD controllers. The graph shows angular displacement (rad) over time (s) for the x-, y-, and z-axes.

The graph shown in Figure 13 illustrates the trajectory tracking performance of the robot end effector under the influence of the two selected control strategies. This result shows the position of the end effector along the x, y, and z axes over time, with the angular displacement measured in radians. Each trajectory is represented by a dashed line; the NTSM controller trajectories are depicted in red, blue, and green, while the PD controller trajectories are shown in black, cyan, and magenta.

### 3.2. Analysis of the Tumor Tissue in the Lung Phantom

This part of the study presents the results of the tumor tissue inserted into the manufactured lung phantom and the image-processing algorithm implemented to calibrate the desired trajectory for the biopsy needle.

#### 3.2.1. Lung Phantom

**Figure 14:**
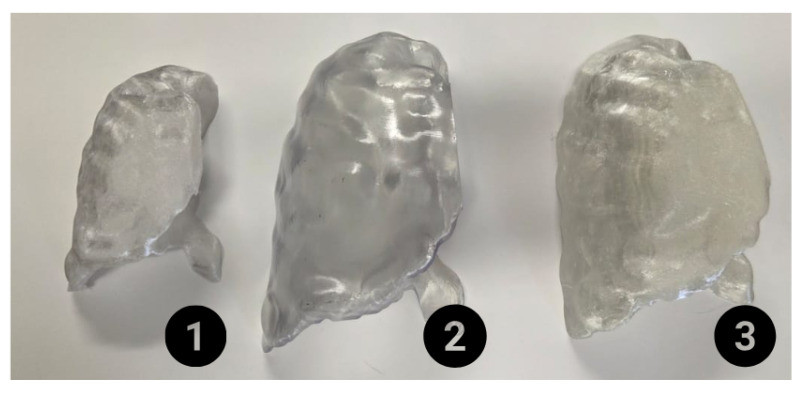
3D-printed lung models were fabricated using various transparent materials, including PETG, resin, and TPU. Model one represents a PETG lung, model two shows a clear-resin lung, and model three shows a TPU lung. Numbers in the figure represent different versions of the printed lung with different sizes.

Figure 14 showcases a set of 3D-printed lung models created using different additive manufacturing materials: clear PETG, clear resin, and clear TPU. Each model was printed to evaluate the material’s transparency and suitability for light transmission, a critical factor for LED illumination and imaging applications. Two criteria were selected to determine the final phantom configuration using an internal photon source intended to emulate an emission-based imaging scenario: source visibility and spatial resolution. These parameters were assessed using images acquired by the camera-based imaging system.

Among the proposed phantoms, the one made of clear resin was selected for controller analysis and CBRS evaluation. This selection was made on the basis of the highest correlation between the geometry of the original photon source and that of the detected photon.

**Figure 15:**
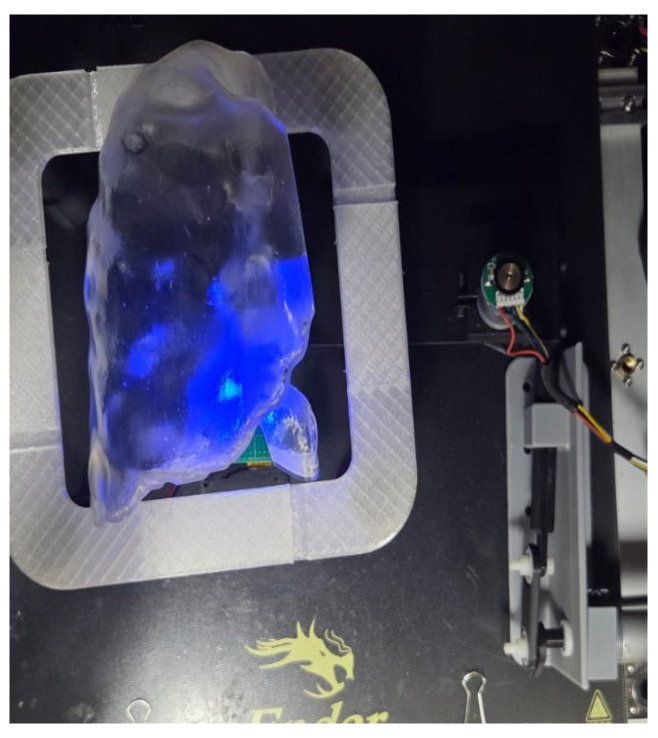
3D lung emulator with simulated tumor illuminated for detection.

Figure 15 illustrates the selected 3D lung phantom incorporating a tumor-like target and an internal simulated emission source. The target is represented by an illuminated region generated using a high-power blue LED to enhance signal visibility within the image-guided framework. This configuration enables the evaluation of image acquisition and segmentation algorithms, allowing for the camera-based detection and centroid localization of the tumor-like target within the lung phantom.

#### 3.2.2. Segmentation and Find the Centroid of the Emulated Tumor Tissue

**Figure 16:**
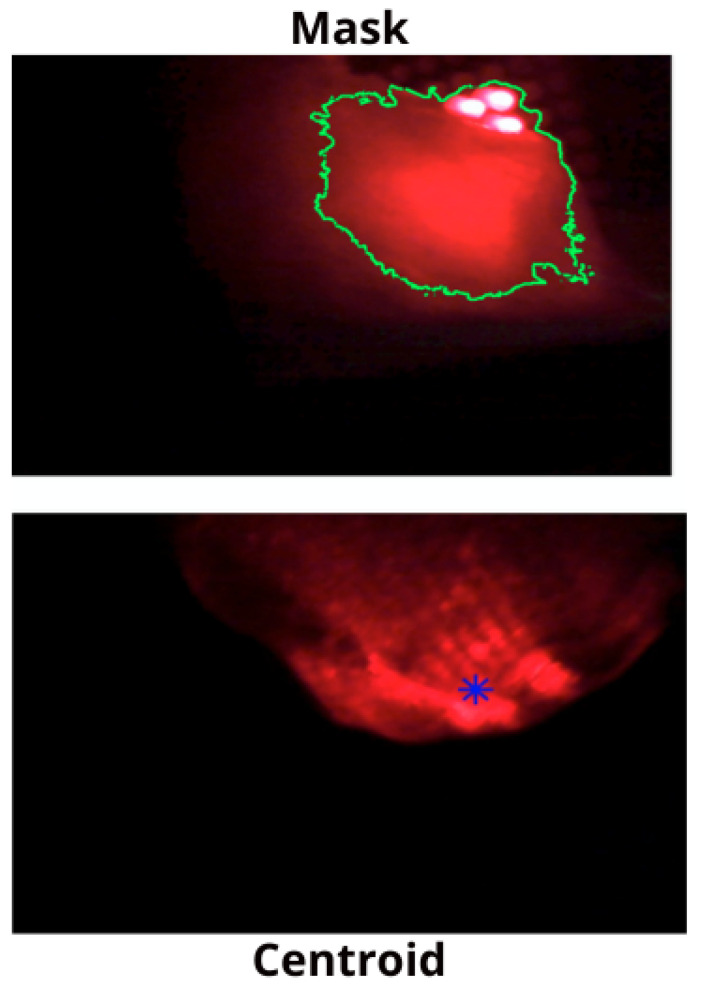
Image segmentation and centroid detection of the tumor.

Figure 16 illustrates the image processing steps for detecting and localizing a tumor within a lung emulator. The top image (Mask) shows the segmented interest region highlighted with a green contour around the illuminated tumor area. The lower image (centroid) shows the detected centroid marked with a blue asterisk, providing a precise location for further analysis or robotic navigation.

**Figure 17:**
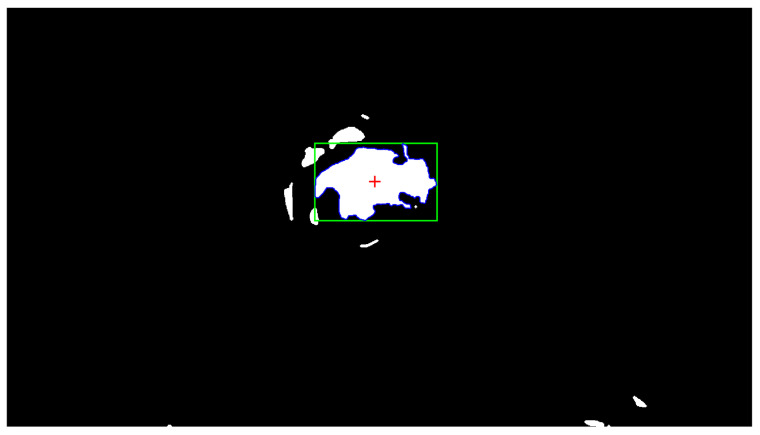
Quantitative visualization of the segmentation pipeline. The binary mask (white) is obtained using Otsu thresholding followed by morphological opening, closing, and hole filling. The largest connected component is highlighted by its bounding box (green), boundary contour (blue), and centroid location (red cross).

These results were obtained using Otsu thresholding followed by morphological post-processing, including opening with a disk structuring element of radius 2 px, closing with a radius of 3 px, and hole filling, and MATLAB image processing as shown in Figure 17.

For each processed frame, the following metrics are computed: (i) the total segmented area,

(29) $$A_{\mathrm{tot}}=\mathrm{nnz}\left(bw\right),$$

expressed in pixels; (ii) the area of the largest connected component,

(30) $$A_{\max}=\max\left({\mathrm{Area}}_{i}\right),$$

obtained via regionprops; and (iii) the centroid coordinates $\left(c_{x},c_{y}\right)$, corresponding to the largest segmented region.

Table 3 reports a representative set of quantitative segmentation metrics. The centroid location, eccentricity, and solidity values indicate stable object localization with limited fragmentation, while the dominant connected component area confirms the correct isolation of the illuminated region.

The LED array consists of five miniature blue LEDs connected in parallel and powered by a 3.7 V source. A fixed protection resistor of 33 Ω is connected in series with a variable resistor *R*, resulting in a total series resistance:

(31) $$R_{\mathrm{tot}}=R+33.$$

Assuming a nominal forward voltage of $V_{f}\approx 3.0$ V, the total current supplied to the LED array is approximated as

(32) $$I_{\mathrm{tot}}=\frac{V_{cc}-V_{f}}{R_{\mathrm{tot}}}.$$

Table 4 presents the estimated total current and the corresponding segmented LED area for different resistance values. The segmented area, expressed in pixel units, is used as a relative proxy for the apparent optical brightness. Both the total current and the segmented area are normalized with respect to the maximum current condition (R=128 Ω).

As the total series resistance decreases, the electrical current increases, following an inverse relationship. However, the optical response estimated from the segmented area exhibits a clearly nonlinear behavior. For example, at approximately 42% of the maximum current, the segmented area already exceeds 50% of its maximum value. This nonlinear response is attributed to camera sensor saturation, blooming effects, and the threshold-based segmentation process.

**Figure 18:**
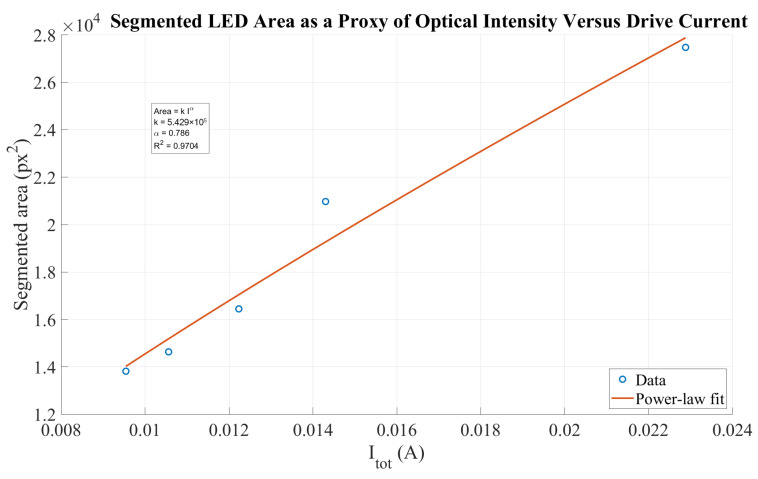
Functional relationship between the total drive current and the segmented LED area. The segmented area (px^2^) is used as a relative proxy of apparent optical brightness.

To further characterize this behavior, the relationship between the segmented area and the total current was modeled using a power-law expression,

(33) $$\mathrm{Area}=5.429\times 10^{5}\;I^{0.786}$$

which provided a high coefficient of determination ($R^{2}=0.9704$), confirming the nonlinear dependence between the electrical drive current and the camera-based optical response as shown in Figure 18.

### 3.3. Integration of Mechanical and Image Systems

**Figure 19:**
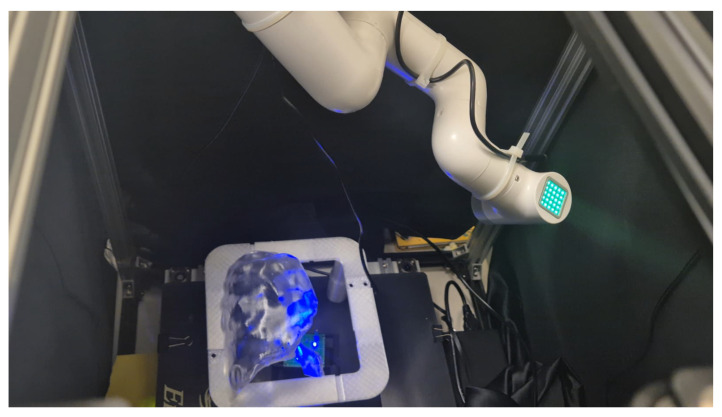
Experimental setup for robotic tumor detection using a lung emulator.

Figure 19 showcases the experimental setup used for robotic tumor detection. For the PD and NTSM controllers, the gains were selected by experimentation. Table 2 summarizes the proportional–derivative (PD) control gains implemented for both the XY Cartesian stage and the MyCobot joints within the digital twin environment. The gains were tuned empirically to ensure stable trajectory tracking and smooth transitions between the planar motion and the robotic arm control.

For the XY stage, the gains $K_{p}=0.8$ and $K_{d}=0.1$ were selected to achieve a maximum steady-state error below 4mm, allowing precise positioning of the target before transfer of control to the manipulator (Table 5).

For the MyCobot arm, diagonal gain matrices $K_{p}^{xyz}=\mathrm{diag}\left(\left[1,1,1\right]\right)$ and $K_{d}^{xyz}=\mathrm{diag}\left(\left[1,1,1\right]\right)$ were used to regulate the motion of the end-effector in Cartesian space, while yaw orientation control was used $K_{p}^{yaw}=0.7$ and $K_{d}^{yaw}=0.20$. These parameters ensure dynamic stability during the execution of the 3D sigmoid trajectory and minimize overshoot in the final alignment phase. On the other hand, Table 6 summarizes the parameters used for the Non-Singular Terminal Sliding Mode (NTSM) controllers implemented in both the Cartesian XY stage and the MyCobot robotic arm. The gains were tuned experimentally to ensure robust convergence, mitigate chattering through a bounded tanh function, and maintain tracking stability within the ±4 mm error tolerance.

#### 3.3.1. Trajectory Tracking Performance Along Each Axis

**Figure 20:**
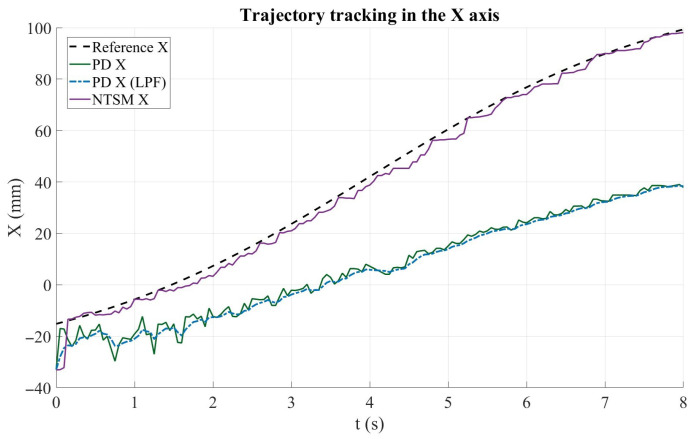
Trajectory tracking performance along the X axis for the PD and NTSM controllers. The reference trajectory (Ref X) is shown in dashed black, PD controller response (PD X) in dark green, filtered PD response (PD $X_{f}$) in blue dashed, and NTSM controller response (NTSM X) in purple.

Figure 20 illustrates the trajectory tracking performance along the X axis for the evaluated control strategies. The dashed black curve represents the reference trajectory, whereas the solid green and dashed blue curves correspond to the PD controller response before and after applying the low-pass filter (LPF), respectively. The purple curve shows the NTSM controller’s response. Overall, the NTSM controller follows the reference trajectory with a smooth response; however, both PD-based strategies exhibit a noticeable steady tracking offset, remaining below the reference path as the motion progresses. Although the LPF attenuates high-frequency oscillations in the PD output, it does not eliminate the tracking bias observed in the X direction.

**Figure 21:**
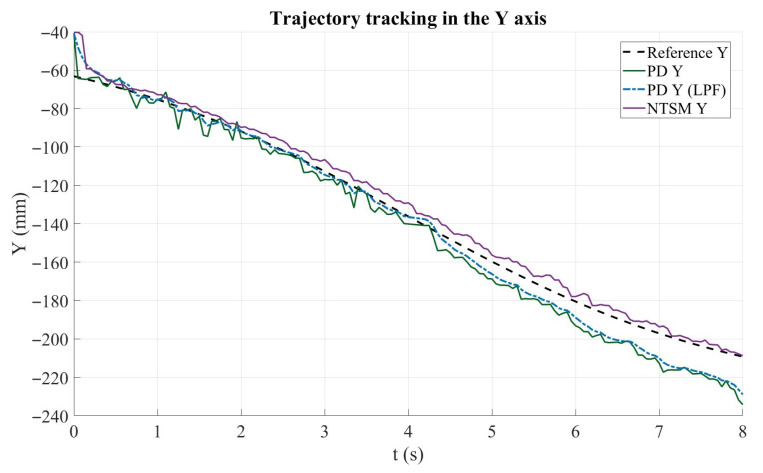
Trajectory tracking performance along the Y axis for the PD and NTSM controllers. The reference trajectory (Ref Y) is shown in dashed black, PD controller response (PD Y) in dark green, filtered PD response ($PDY_{f}$) in blue dashed, and NTSM controller response (NTSM Y) in purple.

Figure 21 shows the trajectory tracking performance along the Y axis. The dashed black curve corresponds to the reference trajectory, whereas the solid green and dashed blue curves represent the PD controller response before and after applying the low-pass filter (LPF), respectively. The purple curve denotes the NTSM controller response. Overall, the NTSM controller follows the reference trajectory more closely throughout the entire motion interval, maintaining smaller deviations and more consistent tracking behavior. In contrast, both PD-based responses exhibit a noticeable steady-state offset, remaining below the reference trajectory as time progresses, which indicates accumulated tracking lag in the Y direction. Although the LPF attenuates small oscillations in the PD response, it does not correct the persistent tracking bias. These results highlight the superior tracking accuracy of the NTSM controller along the Y axis compared to the PD controller, particularly in terms of reducing long-term deviation from the desired reference.

**Figure 22:**
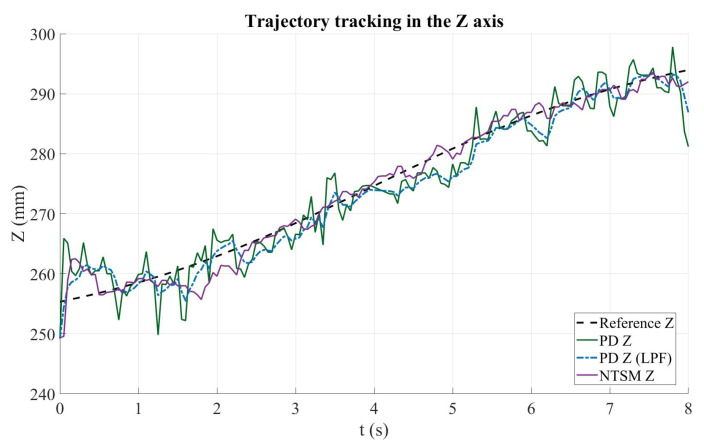
Trajectory tracking performance along the Z axis for the PD and NTSM controllers. The reference trajectory (Ref Z) is shown in dashed black, PD controller response (PD Y) in dark green, filtered PD response ($PDZ_{f}$) in blue dashed, and NTSM controller response (NTSM Z) in purple.

Figure 22 shows the trajectory-tracking performance along the Z axis. The dashed black curve corresponds to the reference trajectory, while the solid green and dashed blue curves represent the PD controller response before and after applying the low-pass filter (LPF), respectively. The purple curve denotes the NTSM controller response. Overall, all controllers follow the increasing Z reference profile with a similar trend across the full motion interval. However, the unfiltered PD response exhibits larger oscillations and sporadic deviations from the reference, particularly during the initial and mid portions of the trajectory, as well as the MP controller variant. Applying the LPF reduces these high-frequency fluctuations, resulting in a smoother PD tracking response. Likewise, the NTSM controller maintains a more regular trajectory evolution and remains closer to the reference during most of the motion, indicating improved robustness against measurement noise and small disturbances. Toward the end of the trajectory, slight deviations are observed in all responses, although the NTSM and filtered PD outputs maintain closer agreement with the reference than in the unfiltered PD case.

To track the trajectory along each axis, it’s notable that the NTSM controller produces a smoother response relative to the reference, reducing steady-state error, avoiding deviations, and allowing larger displacements. The NTSM control scheme outperforms the PD controller across all three axes, providing faster convergence and better robustness to dynamic variations.

#### 3.3.2. Tracking Error Along Each Axis

**Figure 23:**
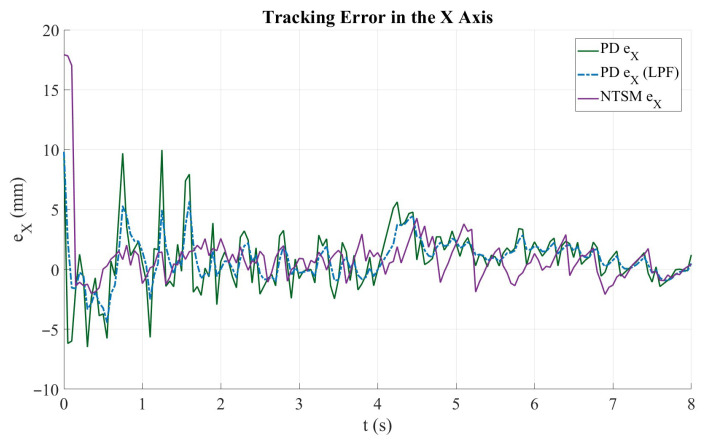
Tracking error along the X axis for the PD and NTSM controllers. The plot compares the instantaneous error of the PD controller (PD $e_{x}$), its filtered output (PD $e_{x_{f}}$), and the NTSM controller (NTSM $e_{x}$).

Figure 23 shows the time evolution of the tracking error along the X axis for both control schemes. The solid green curve corresponds to the PD controller error, the dashed blue curve represents the PD error after applying the low-pass filter (LPF), and the purple curve denotes the NTSM controller error. During the initial stage of the motion (t<1 s), both controllers exhibit a transient response with pronounced peaks caused by the abrupt trajectory start-up, with the NTSM error reaching the largest initial overshoot. After this transient, the PD controller presents higher-frequency oscillations and intermittent spikes, which are significantly attenuated by the LPF, leading to a smoother error evolution. In contrast, the NTSM controller provides a comparatively smoother profile during most of the trajectory, with reduced high-frequency fluctuations and a bounded error magnitude. Overall, both strategies achieve comparable tracking performance along the X axis; however, the filtered PD response improves stability by reducing oscillations, while the NTSM controller maintains a more regular error evolution across the motion. Quantitatively, the X-axis RMSE increases slightly from 2.69 mm (PD) to 2.78 mm (NTSM), corresponding to a 3.32% degradation, indicating comparable tracking performance between the two strategies along this axis.

**Figure 24:**
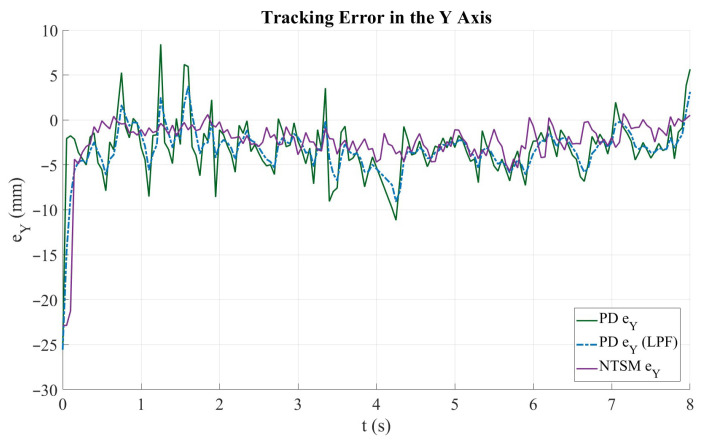
Tracking error along the Y axis for the PD and NTSM controllers. The plot compares the instantaneous error of the PD controller (PD $e_{y}$), its filtered output (PD $e_{y_{f}}$), and the NTSM controller (NTSM $e_{y}$).

Figure 24 illustrates the tracking error evolution along the Y axis for the evaluated control strategies. The solid green curve corresponds to the PD controller error, the dashed blue curve represents the PD error after applying a low-pass filter (LPF), and the purple curve denotes the NTSM controller error. At the beginning of the motion (t<1 s), a pronounced transient response is observed, characterized by a negative peak close to −25 mm, which is mainly associated with the abrupt trajectory start-up and the initial adjustment to the reference signal. After this transient, the PD controller exhibits higher-frequency oscillations and occasional spikes, indicating sensitivity to measurement noise and external disturbances; these oscillations are partially attenuated by the LPF, resulting in a smoother error profile. In contrast, the NTSM controller provides smoother, more bounded error evolution throughout most of the trajectory, with reduced high-frequency fluctuations and improved steady-state stability. Overall, both strategies maintain stable tracking along the Y axis; however, the NTSM controller exhibits more consistent error behavior and lower long-term deviation than the PD controller. Quantitatively, the Y-axis RMSE decreases from 4.61 mm (PD) to 3.90 mm (NTSM), corresponding to a 15.26% improvement.

**Figure 25:**
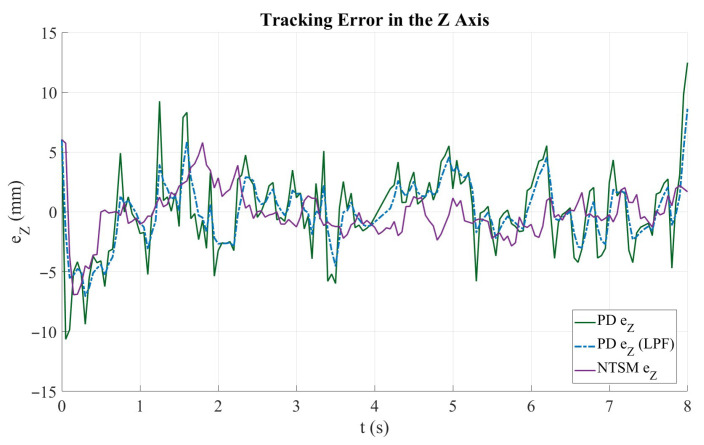
Tracking error along the Z axis for the PD and NTSM controllers. The plot compares the instantaneous error of the PD controller (PD $e_{z}$), its filtered output (PD $e_{z_{f}}$), and the NTSM controller (NTSM $e_{z}$).

Figure 25 shows the evolution of tracking error along the Z axis for the evaluated control strategies. The solid green curve corresponds to the PD controller error, the dashed blue curve represents the PD error after applying a low-pass filter (LPF), and the purple curve denotes the NTSM controller error. At the beginning of the motion (t<1 s), both controllers exhibit a transient response with pronounced peaks, reaching approximately −10 mm for the unfiltered PD case, while the filtered PD and NTSM responses remain within a narrower range. During the remaining trajectory, the PD controller shows higher-frequency oscillations and intermittent spikes, which are noticeably attenuated by the LPF, resulting in a smoother error evolution. In contrast, the NTSM controller yields a smoother error profile with reduced high-frequency fluctuations, although a small steady-state offset can be observed in specific intervals. Toward the end of the experiment (t≈8 s), a sharp increase in the error is observed, particularly in the PD-based response, indicating reduced stability under the final portion of the trajectory. Overall, the NTSM controller provides a more stable, bounded evolution of tracking error along the Z axis compared to the PD controller. Quantitatively, the Z-axis RMSE decreases from 3.54 mm (PD) to 1.99 mm (NTSM), corresponding to a 43.86% improvement.

We extended our MATLAB analysis code to automatically compute quantitative statistics for each run, including the mean error (RMSE), maximum absolute error (MAE), and standard deviation for eX, eY, eZ, and e3D (in mm). In addition, we quantify convergence by computing a settling time defined as the first time instant at which e3D(t) remains below an absolute threshold ϵ=3 mm for at least $T_{\mathrm{hold}}=0.20$ s. We then report the mean and standard deviation of the settling time across runs for PD and NTSM.

Table 7 reports the aggregated quantitative metrics (mean, standard deviation, and maximum across runs) for both controllers, including the settling-time statistics under the criterion $e_{3D}\leq 3.0$ mm for at least 0.20 s.

**Figure 26:**
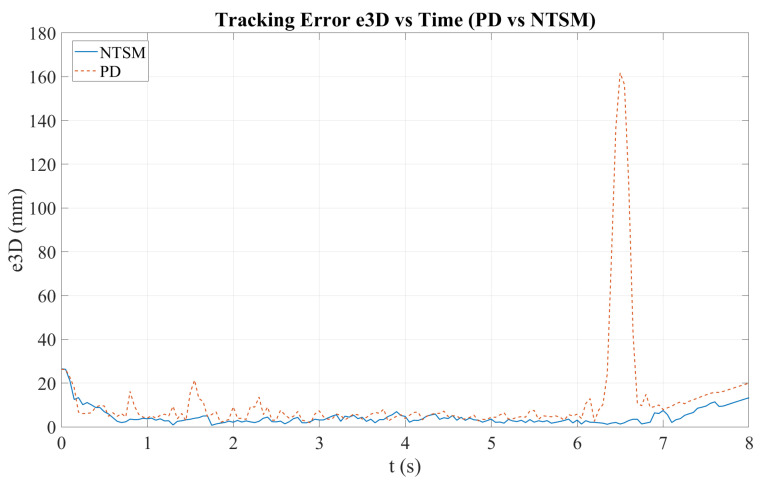
Tracking error in 3D (e3D) versus time for PD and NTSM.

Figure 26 shows the time evolution of the 3D tracking error (e3D) for both controllers. Although both methods reduce the initial transient error, the NTSM controller maintains a more stable behavior with smaller fluctuations over time. In contrast, the PD controller exhibits higher variability and a pronounced transient peak, which substantially increases the maximum absolute error and negatively impacts overall tracking consistency.

**Figure 27:**
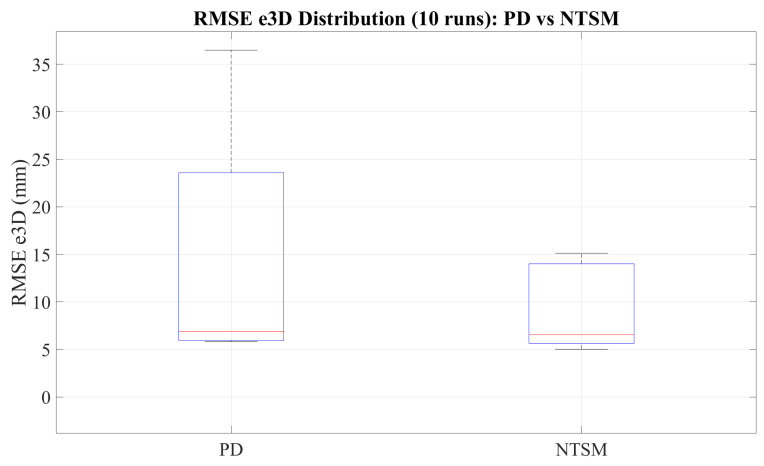
RMSE e3D distribution across 10 runs for PD and NTSM.

Figure 27 summarizes the tracking accuracy by comparing the RMSE e3D distributions across 10 independent runs for both controllers. The NTSM controller exhibits a lower median RMSE and a narrower interquartile range than the PD controller, indicating improved repeatability and reduced variability across trials. In contrast, the PD controller shows greater dispersion and higher extreme values, which increase the overall error distribution and reflect greater sensitivity to disturbances and trial-to-trial variations.

**Figure 28:**
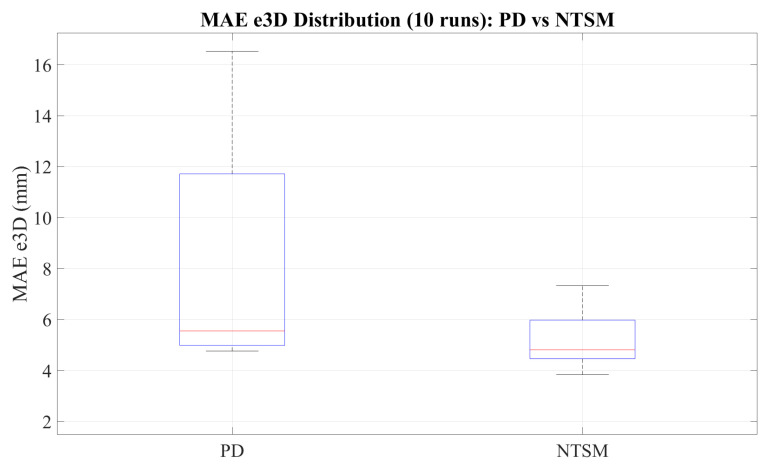
MAE e3D distribution across 10 runs: PD vs. NTSM.

Figure 28 compares the distribution of the 3D mean absolute error (MAE e3D) across 10 independent runs for both controllers. The NTSM controller exhibits a lower MAE e3D and a tighter distribution than the PD controller, indicating improved tracking consistency and repeatability. In contrast, the PD controller shows larger dispersion and higher extreme values, reflecting increased sensitivity to disturbances and greater run-to-run variability.

**Figure 29:**
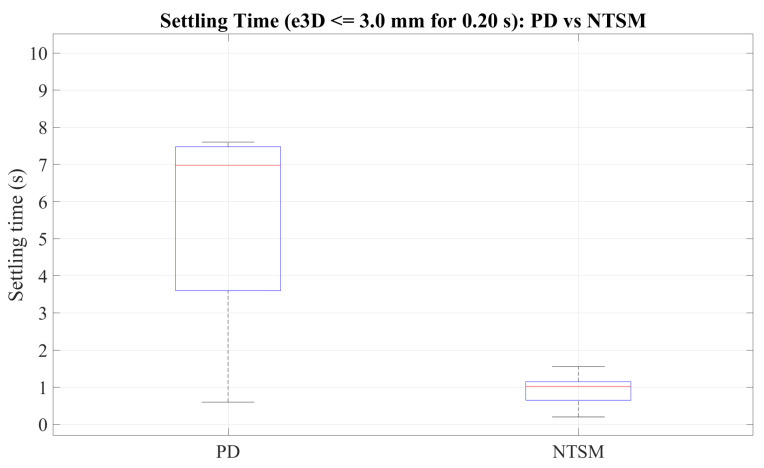
Settling time comparison between PD and NTSM using the criterion $e_{3D}\leq 3.0$ mm sustained for 0.20 s.

Figure 29 reports the settling time distributions obtained for PD and NTSM based on the convergence criterion $e_{3D}\leq 3.0$ mm maintained for 0.20 s. The NTSM controller reaches the steady-state error bound substantially faster than PD, with a markedly lower median settling time and a narrower interquartile range. In contrast, PD exhibits significantly longer convergence times and larger dispersion across runs, indicating lower repeatability and higher sensitivity to transient disturbances.

#### 3.3.3. Three-Dimensional End-Effector Trajectory Tracking and Tracking Error

**Figure 30:**
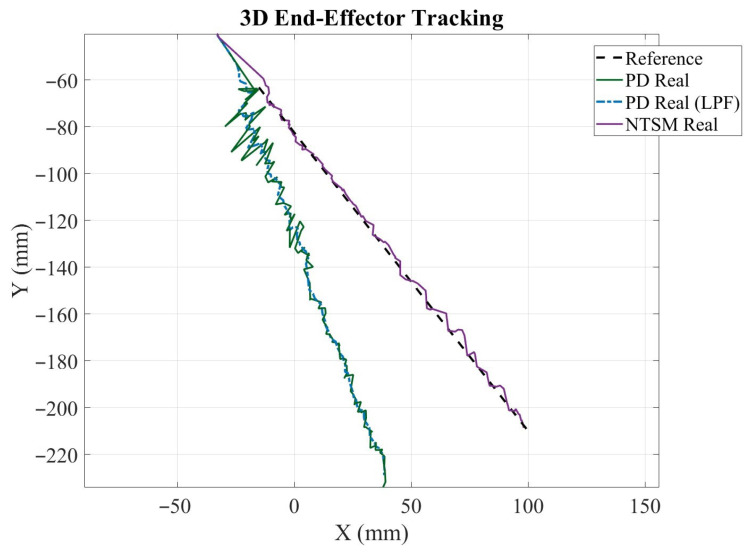
3D end-effector tracking in the XY plane: reference trajectory (dashed black) versus measured trajectories for PD (raw), PD with low-pass filtering (PD Real (LPF)), and NTSM.

Figure 30 illustrates the end-effector tracking performance under both control schemes compared to the desired reference path in the Cartesian space. The dashed black line represents the reference trajectory, while the solid green and dashed blue curves correspond to the PD controller output before and after low-pass filtering (LPF), respectively. The purple curve denotes the trajectory achieved by the NTSM controller.

Although both controllers follow the overall direction of the reference path, clear differences in tracking accuracy are observed. The PD controller exhibits larger deviations from the reference, particularly as the trajectory progresses toward more negative *Y* values, showing a noticeable offset and increased dispersion around the desired path. The LPF version of PD slightly smooths the trajectory but does not eliminate the systematic deviation. In contrast, the NTSM controller remains closer to the reference curve along most of the motion, with smaller spatial discrepancies and improved consistency. Overall, these results indicate that NTSM provides enhanced tracking accuracy and robustness compared to PD under the evaluated conditions.

**Figure 31:**
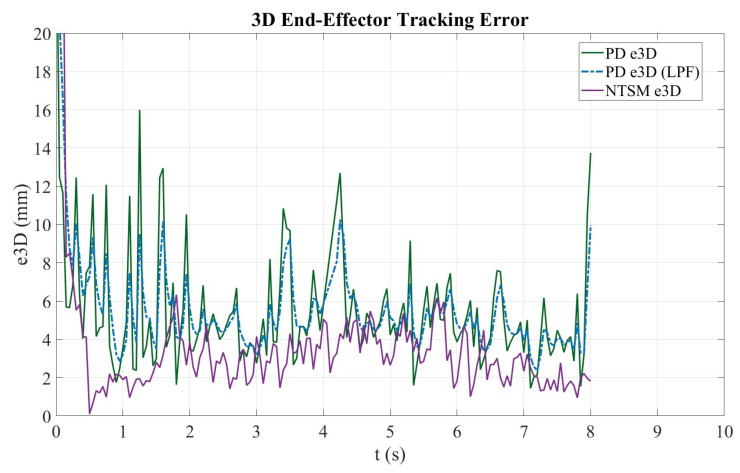
3D end-effector tracking error ($e_{3D}$) versus time for PD (raw), PD with low-pass filtering (PD $e_{3D}$ (LPF)), and NTSM.

Adding to this, Figure 31 illustrates the evolution of the 3D tracking error ($e_{3D}$) of the robotic end-effector for both control schemes. The solid green and dashed blue curves correspond to the PD controller before and after low-pass filtering (LPF), respectively, while the purple curve represents the NTSM controller.

At the beginning of the trajectory (t<0.5 s), both controllers exhibit a transient peak near 20 mm, which is mainly due to the initial positioning adjustment and controller activation. After this initial transient, the NTSM controller quickly reduces the tracking error and maintains a consistently lower level, typically around 1–4 mm for most of the experiment. In contrast, the PD controller shows larger oscillations and greater variability, with frequent peaks of approximately 8–12 mm and occasional higher spikes at specific instants. The LPF version of PD effectively attenuates high-frequency oscillations and smooths the error signal, but it does not fully remove the larger deviations observed in the raw PD response. Overall, the results indicate that NTSM achieves improved stability and tracking consistency compared to PD, maintaining a lower, more uniform $e_{3D}$ profile throughout the trajectory.

Overall, both controllers achieve acceptable 3D tracking accuracy; however, the NTSM controller demonstrates greater robustness and stability in the transient response, while the PD controller achieves slightly better steady-state precision under ideal conditions.

### 3.4. Hysteretic Reference Trajectory for Lung Emulation and Control Assessment

To ensure proper emulation of lung motion and to assess controller performance, encoder counts were recorded throughout the hysteretic motion cycle. The controller gains were empirically tuned through experiments, yielding $K_{p}=7.0$ and $K_{d}=0.15$, which ensured stable operation and satisfactory trajectory tracking.

**Figure 32:**
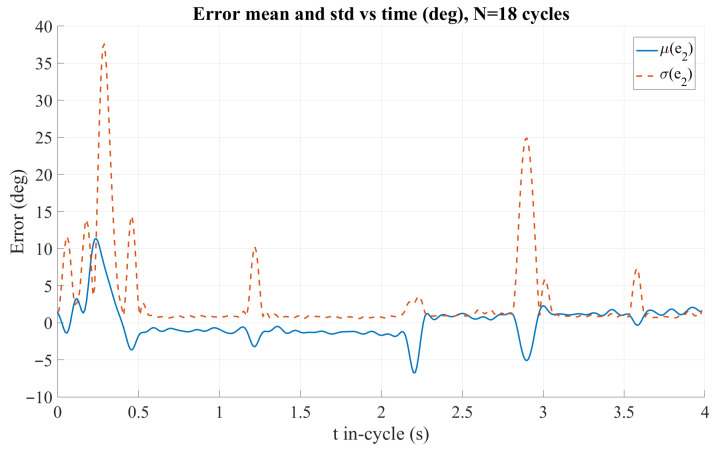
Motor 2 tracking error statistics in degrees over one motion cycle. The solid line shows the mean error $\mu (e_{2})$, and the dashed line shows the standard deviation $\sigma (e_{2})$, computed across N=18 cycles.

Figure 32 presents the low-pass filtered (LPF) tracking error statistics of Motor 2 in degrees over one motion cycle, computed across N=18 repetitions. The solid curve corresponds to the mean error, $\mu (e_{2})$, whereas the dashed curve represents the standard deviation, $\sigma (e_{2})$. Overall, the mean error remains close to 0deg during most of the cycle, indicating a small steady tracking bias. However, transient deviations are observed at the beginning of the motion (t<0.5s) and near the trajectory transition region (t≈2.2–3.0 s), where the mean error exhibits short negative dips. The cycle-to-cycle variability is generally low, but pronounced peaks in $\sigma (e_{2})$ appear at specific instants, particularly around t≈0.3s and t≈2.9s, suggesting intermittent disturbances or inconsistent tracking behavior concentrated in those intervals.

**Figure 33:**
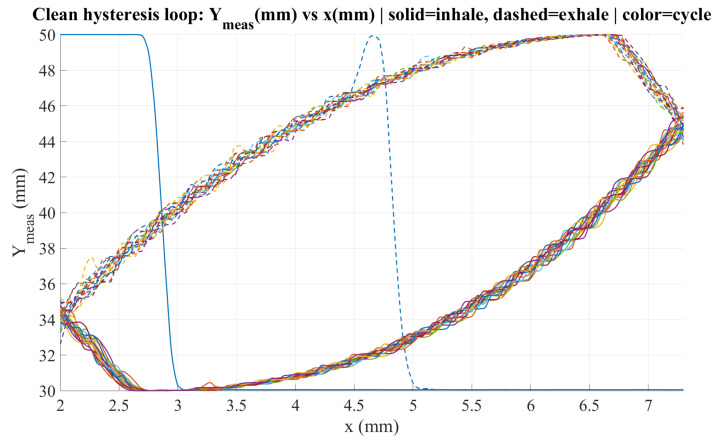
Clean hysteresis loop obtained from the measured displacement $Y_{\mathrm{meas}}$ (mm) as a function of the horizontal position *x* (mm) over multiple cycles. Solid curves correspond to the inhalation phase, while dashed curves represent the exhalation phase. Each color denotes a different cycle, highlighting the repeatability of the hysteresis behavior and the cycle-to-cycle dispersion along the trajectory.

Figure 33 illustrates the clean hysteresis loop obtained by plotting the measured displacement $Y_{\mathrm{meas}}$ (mm) as a function of the horizontal position *x* (mm) over multiple cycles. The solid curves correspond to the inhalation phase, whereas the dashed curves represent the exhalation phase, and different colors indicate different repetitions. The results show a consistent hysteresis pattern, with the system following distinct trajectories during inhalation and exhalation at the same *x* position, confirming path-dependent behavior. Overall, the curves remain closely clustered across cycles, indicating good repeatability; nevertheless, small cycle-to-cycle deviations are observed mainly near the turning regions, which may be associated with transient dynamics, backlash, or measurement noise.

### 3.5. Robustness Analysis of the Controller Implementation

**Figure 34:**
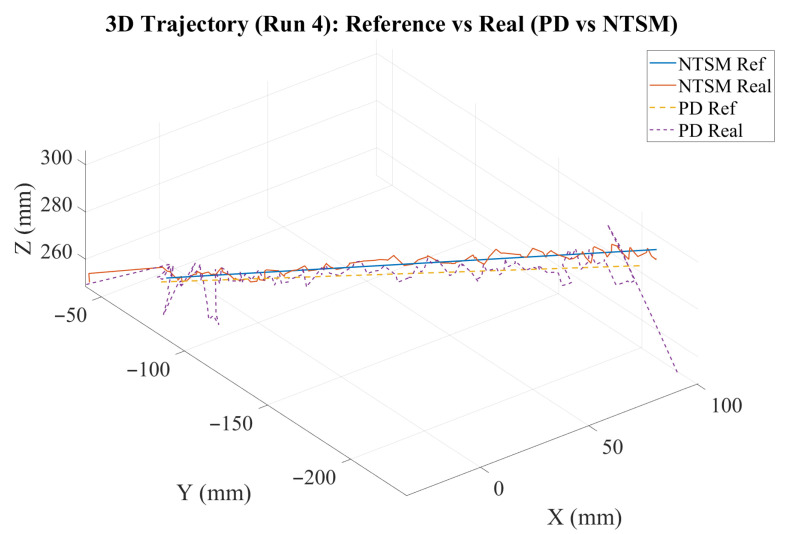
3D trajectory comparison (Run 4, +30 g payload): reference vs. measured trajectories for PD and NTSM.

**Figure 35:**
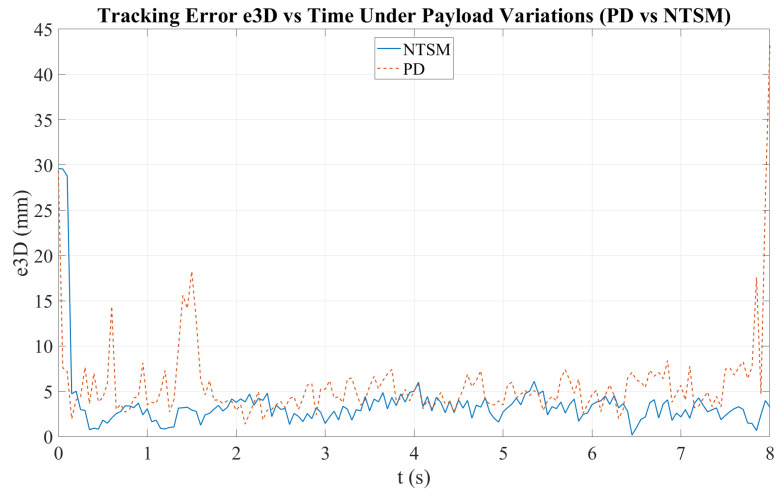
Tracking error in 3D (e3D) versus time under payload variations: PD vs. NTSM.

**Figure 36:**
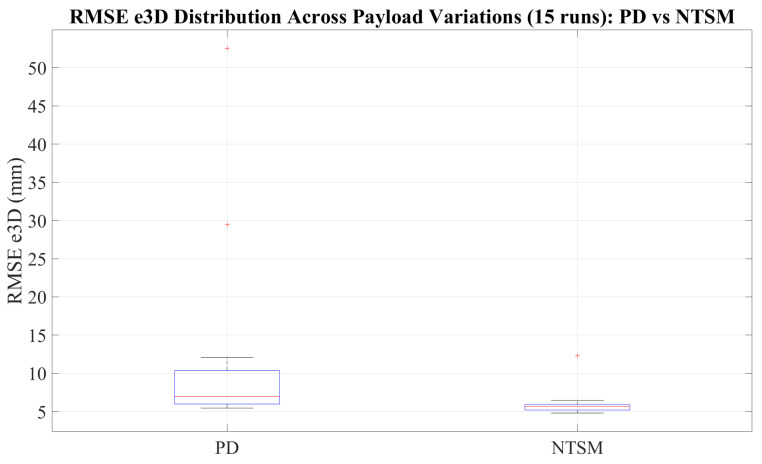
RMSE e3D distribution across payload variations (15 runs): PD vs. NTSM.

**Figure 37:**
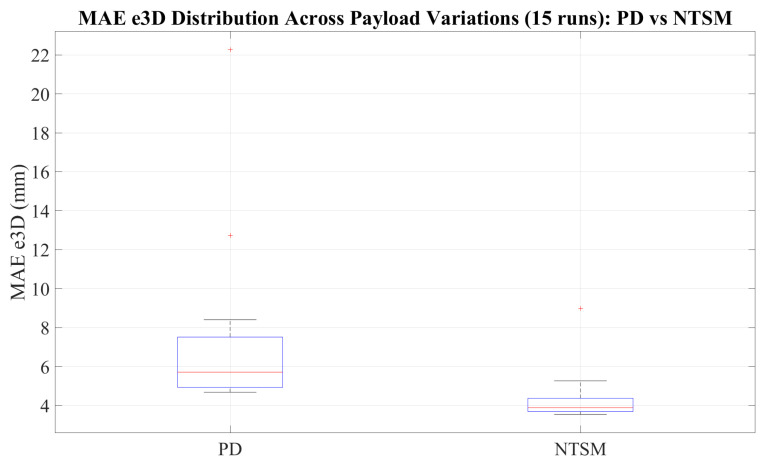
MAE e3D distribution across payload variations (15 runs): PD vs. NTSM.

The robustness analysis of the proposed controller uses a mass attached to the end-effector of the manipulator and the corresponding tracking evaluation. To perform this analysis, a custom box with an initial weight of 140 g was manufactured and mounted on the robot, and additional weights of 10 g, 30 g, and 50 g were progressively added to evaluate the algorithm under different payload conditions. For each loading scenario, five trials were performed to assess repeatability, resulting in a total of 15 runs. The experimental results demonstrate improved performance of the NTSM controller compared to the PD controller, as shown in Figure 34, Figure 35, Figure 36 and Figure 37.

Figure 34 shows the 3D reference and measured trajectories for Run 4, which corresponds to the +30 g payload condition. Under this load, the NTSM controller maintains a trajectory closer to the reference path, whereas the PD controller exhibits larger deviations and higher fluctuations. These results indicate that NTSM provides improved tracking accuracy and enhanced robustness against payload-induced disturbances.

Figure 35 presents the time-domain evolution of the 3D tracking error (e3D) under payload variations. The NTSM controller achieves a more stable tracking response, with reduced error magnitude and fewer fluctuations throughout the motion. In contrast, the PD controller shows higher variability and more pronounced transient peaks, suggesting lower robustness to load disturbances.

Figure 36 summarizes the overall tracking accuracy through the distribution of RMSE e3D across all experimental trials under varying payload conditions. The boxplot comparison shows that the NTSM controller yields a lower median RMSE e3D and a narrower interquartile range than the PD controller, reflecting improved repeatability and robustness under load changes. Moreover, PD exhibits larger dispersion and more pronounced outliers.

Figure 37 compares the distribution of the 3D mean absolute error (MAE e3D) across all trials. The results show that the NTSM controller achieves consistently lower MAE e3D and reduced variability compared to the PD controller, indicating improved tracking consistency under external load conditions.

## 4. Discussion

Manual lung biopsy procedures remain a critical, yet challenging component of cancer diagnosis. Key challenges include the need for high-precision targeting of cancerous tissues, the influence of respiratory motion on tissue positioning, and the potential for human error during intervention. Traditional approaches rely heavily on the expertise of the surgeon and real-time imaging techniques, such as CT scans, to guide biopsy needle placement. However, these methods are prone to inaccuracies, particularly in the presence of lung motion, leading to misalignment between the tumor and the biopsy needle.

In contrast, the proposed system addresses these challenges by integrating a multi-degree-of-freedom robotic manipulator with an autonomous machine learning-based video processing algorithm. The key advantage of this approach lies in its ability to autonomously adjust the biopsy needle based on real-time tumor location data, minimizing the influence of motion and reducing the likelihood of human error.

A major advancement of the proposed system is the use of machine learning to detect and track tumors in real-time. Traditional methods, such as CT imaging, provide static information that can be prone to inaccuracies due to patient movement, respiratory cycles, or slight misalignments between the imaging data and the biopsy needle. The integration of image processing in this study enables dynamic, adaptive tumor detection, with the system continually updating the tumor’s location using video data from a lung motion emulator and a photon emitter.

Although traditional imaging remains a cornerstone of current biopsy techniques, image processing offers significant advantages in terms of adaptability, accuracy, and speed. By providing continuous feedback on tumor position and boundaries, the image processing model in this study enhances the precision of the robotic manipulator, ensuring that the needle is accurately directed even under dynamic conditions.

Current robotic biopsy systems often rely on preprogrammed motion paths or manual input from the operator to guide the needle toward the target. These systems are typically designed to operate in controlled environments, where patient movement is minimal and lung motion is either ignored or compensated for through mechanical adjustments. However, this approach may still suffer from limitations in precision and response time, particularly in cases involving rapid or irregular motion.

In contrast, the proposed system incorporates a finite-time convergent controller for the robotic manipulator, ensuring accurate needle placement even in dynamic conditions. By tracking the centroid coordinates of the tumor and adjusting the robotic arm’s trajectory in real-time, the system compensates for any changes in the target’s position, thereby improving the robustness and reliability of the procedure. The successful tracking of the reference trajectories, validated through a digital twin and a physical mobile tumor emulator, confirms the proposed controller’s ability to maintain high precision despite the inherent challenges posed by lung motion.

The system validation was performed using both a digital twin model and a physical mobile tumor emulator, which provides a strong evidence base for its efficacy. Digital twins, which provide highly accurate simulations of the robotic system and tumor movement, enable extensive testing and optimization of the algorithm in a controlled virtual environment. Meanwhile, the use of a physical mobile tumor emulator ensures that real-world variables—such as mechanical tolerance and response times—are thoroughly considered.

This experimental approach not only demonstrates the technical feasibility of the system, but also provides a comprehensive evaluation under varied conditions. The successful targeting and placement of the biopsy needle observed in these tests suggest that the proposed system outperforms traditional manual methods, which lack real-time feedback on tumor positioning during the procedure.

This research has contributed to the design of an autonomous biopsy sampler, including an image processing algorithm to detect the online location of the target tissue. Even though this study has contributed to the development of robotic biopsy samplers, it is noticeable that some open areas still remain as research opportunities; however, these are limitations of our current studies: (a) the robotic device is constructed using an additive manufacturing process, which helped us to simplify the device construction, but this cannot be considered for clinical applications due to the polymeric (porous) materials; (b) the photon source associated with the soft tissue is in the visible range, as is the detector, but this must be tested using gamma sources and the corresponding camera; and (c) the motion velocity of the robotic device can be improved to oversample the photon source, yielding more precise motion tracking, with the eventual inclusion of a camera with higher resolution.

## 5. Conclusions

This study successfully demonstrates the design and implementation of an autonomous video processing algorithm integrated with a multi-degree-of-freedom robotic manipulator for performing lung biopsies. The proposed system offers a promising solution to the challenges faced in manual biopsy procedures, particularly with respect to tissue movement and the precision required to locate and target cancerous tissue. Through the use of a realistic lung motion emulator and a photon emitter for radiopharmaceutical identification, the system effectively detects tumor boundaries and centroid coordinates, which serve as the basis for guiding the biopsy needle to the target.

Integration of image processing for tumor detection and the application of a finite-time convergent controller for robotic motion ensure accurate and timely execution of biopsy tasks. The experimental validation, performed using both a digital twin of the robotic system and a physical mobile tumor emulator, confirms the efficacy of the proposed system in detecting and targeting tumor sites with high precision. Successful tracking of reference trajectories further highlights the robustness and reliability of the controller in dynamic conditions.

This research advances robotic-assisted biopsy procedures, contributing to the automation of cancer diagnosis with improved accuracy and reduced human error. Although the proposed system represents a significant advancement in robotic-assisted biopsy procedures, its application in clinical settings is an area that deserves further exploration. The integration of additional sensory modalities, such as ultrasound or MRI, could potentially enhance the real-time decision-making capabilities of the system, allowing for even more precise targeting in complex anatomical structures. In addition, clinical validation in real-world scenarios involving live patients remains an essential next step. Although the results obtained from simulation-based testing are promising, the system must be rigorously evaluated in clinical trials to confirm its safety, efficacy, and practical applicability in various medical settings. The future integration of such systems into clinical workflows could streamline cancer diagnosis, reduce procedure time, and minimize complications associated with manual biopsy.

## Figures and Tables

**Table 1:** Comparison of clear 3D-printing materials for image-guided phantom fabrication.

Material	Optical Transparency	Advantages for Image-Guided Phantom
Clear PLA	Low-moderate (translucent; layer scattering)	Easy to print; low cost; good dimensional stability
Clear PETG	Moderate-high (more transparent than PLA)	Better light transmission; tougher than PLA; widely available
Clear Resin	High (near-optical clarity)	Excellent transparency; smooth surfaces; high spatial fidelity
Clear TPU	Low-moderate (translucent)	Adds limited flexibility; impact resistant

**Table 2:** Controller gains for PD and NTSM schemes applied to the XY stage and MyCobot joints in the digital twin environment.

Robot Joint	$K_{p}$ (PD)	$K_{d}$ (PD)	$K_{j}$ (NTSM)
X	80	15	15
Y	10,000	10,000	70
J1	1000	2	5
J2	500	15	3
J3	1200	0	2.6
J4	100	5	1.9
J5	10	2	0.5
J6	120	50	0.01

**Table 3:** Representative segmentation metrics obtained from the proposed pipeline.

Metric	Value	Units
Total segmented area $A_{\mathrm{tot}}$	20,974	px
Largest component area $A_{\max}$	16,154	px
Centroid $c_{x}$	632.5322	px
Centroid $c_{y}$	298.9297	px
Bounding box (x,y,w,h)	(529.5, 232.5, 232.5, 209)	px
Perimeter	774.5480	px
Eccentricity	0.8255	–
Solidity	0.7917	–

**Table 4:** Estimated total current and segmented LED area for different resistance values. The last configuration (R=128 Ω) is used as the 100% reference.

*R* (Ω)	$R_{\mathrm{tot}}$ (Ω)	Area (px^2^)	$I_{\mathrm{tot}}$ (A)	% $I_{\mathrm{tot}}$	% Area
354	387.7	13,816	0.00954	41.7	50.3
316.6	350.3	14,633	0.01056	46.2	53.2
268.7	302.4	16,445	0.01224	53.5	59.8
225	258.7	20,974	0.01430	62.5	76.3
128	161.7	27,476	0.02288	100.0	100.0

**Table 5:** PD controller gains for the XY stage and MyCobot arm.

Subsystem	Axis/Parameter	$K_{p}$	$K_{d}$
XY Cartesian stage	XY plane	0.8	0.1
MyCobot (Cartesian space)	[X,Y,Z]	1.0	1.0
	Yaw (ψ)	0.7	0.20

**Table 6:** NTSM controller parameters for the XY stage and MyCobot arm.

Symbol	Description	XY Stage	MyCobot Arm
α	Power coefficient	0.5	0.5
β	Nonlinear exponent	1.1	1.2
γ	Fractional exponent	0.5	0.5
*c*	Surface gain term	0.9	0.8
λ	Derivative weight	0.02	0.02
$k_{1}$	Reaching gain	5.0	4.0
$k_{2}$	${\left|s\right|}^{\gamma }$ gain	1.0	1.0
ϕ	Boundary layer (mm)	3.0	2.0

**Table 7:** Quantitative comparison between PD and NTSM across 10 runs. Reported values correspond to mean ± standard deviation (Std), and maximum (Max) across runs. Errors are expressed in mm and settling time ($e_{3D}\leq 3$ mm for 0.20 s) in s.

Metric	PD	NTSM	Unit
RMSE ($e_{3D}$)	13.036±11.151 (36.475)	8.619±4.077 (15.119)	mm
MAE ($e_{3D}$)	7.773±4.180 (16.523)	5.191±1.130 (7.336)	mm
MaxAbs ($e_{3D}$)	73.193±77.452 (233.34)	60.654±56.024 (165.95)	mm
Settling time	5.538±3.319 (7.60)	0.935±0.398 (1.55)	s

## Data Availability

Link to the GitHub https://github.com/mondiaz19/Article-Regulating-Automatic-Robotic-Lung-Biopsy-Sampler/issues, accessed on 15 December 2025.
